# Multi-Temporal Effects of Urban Forms and Functions on Urban Heat Islands Based on Local Climate Zone Classification

**DOI:** 10.3390/ijerph16122140

**Published:** 2019-06-17

**Authors:** Jinling Quan

**Affiliations:** State Key Laboratory of Resources and Environmental Information System, Institute of Geographic Sciences and Natural Resources Research, Chinese Academy of Sciences, Beijing 100101, China; quanjl@lreis.ac.cn; Tel.: +86-10-6488-9015

**Keywords:** urban heat island, land surface temperature, local climate zone, remote sensing, Beijing

## Abstract

Urban forms and functions have critical impacts on urban heat islands (UHIs). The concept of a “local climate zone” (LCZ) provides a standard and objective protocol for characterizing urban forms and functions, which has been used to link urban settings with UHIs. However, only a few structure types and surface cover properties are included under the same climate background or only one or two time scales are considered with a high spatial resolution. This study assesses multi-temporal land surface temperature (LST) characteristics across 18 different LCZ types in Beijing, China, from July 2017 to June 2018. A geographic information system-based method is employed to classify LCZs based on five morphological and coverage indicators derived from a city street map and Landsat images, and a spatiotemporal fusion model is adopted to generate hourly 100-m LSTs by blending Landsat, Moderate Resolution Imaging Spectroradiometer (MODIS), and FengYun-2F LSTs. Then, annual and diurnal cycle parameters and heat island and cool island (HI or CI) frequency are linked to LCZs at annual, seasonal, monthly, and diurnal scales. Results indicate that: (1) the warmest zones are compact and mid and low-rise built-up areas, while the coolest zones are water and vegetated types; (2) compact and open high-rise built-up areas and vegetated types have seasonal thermal patterns but with different causes; (3) diurnal temperature ranges are the highest for compact and large low-rise settings but the lowest for water and dense or scattered trees; and (4) HIs are the most frequent summertime and daytime events, while CIs occur primarily during winter days, making them more or less frequent for open or compact and high- or low-rise built-up areas. Overall, the distinguishable LSTs or UHIs between LCZs are closely associated with the structure and coverage properties. Factors such as geolocation, climate, and layout also interfere with the thermal behavior. This study provides comprehensive information on how different urban forms and functions are related to LST variations at different time scales, which supports urban thermal regulation through urban design.

## 1. Introduction

Rapid urbanization and intense human activities have significantly changed the land surface and atmospheric conditions, leading to a series of urban environmental problems worldwide, including urban heat islands (UHIs), which refer to higher urban temperatures compared to surrounding rural ones [1,2,3,4,5]. Numerous studies have been conducted on UHIs considering their social, economic, and environmental impacts (e.g., on human health, energy consumption, air pollution, and climate change) [3,4,5,6,7,8], and main driving factors of UHIs are widely recognized, including, for example, land use and land cover, urban geometry, building materials, landscape patterns, and human activities [4,5,9,10,11]. However, due to non-standardized quantification of the metadata to describe different urban and rural forms and functions, the comparison and communication of the findings and their applications in climatology and urban planning have been greatly limited [12,13].

To address this problem, a local climate zone (LCZ) scheme has been developed to characterize the urban and rural forms and functions under a standard, objective, and quantitative protocol. An LCZ is an area of uniform surface structure, land cover, construction material, and metabolic activity at a minimum radius of 200–500 m with a characteristic screen-height temperature regime [13]. The standard LCZ set defines ten built-up (LCZ 1–10) and seven land cover (LCZ A–G) types with a list of indicator ranges [13] (Table A1). The LCZ system offers a package of 10 basic descriptors of the urban forms and functions (e.g., building height and coverage, pervious and impervious coverage, and aspect ratio) and allows studies on the three-dimensional urban morphology and two-dimensional surface cover in an integrated form. It further strengthens the inter-comparison of UHI studies, bridges the urban fabric and local climate, and provides simple and reliable guidance for the urban planning and management process [13].

Previous studies have adopted the LCZ system to link different urban settings with air temperatures (ATs) and UHIs measured at fixed sites and vehicle traverses, or simulated by numerical models in cities, such as Hong Kong, China [14], Dublin, Ireland [15], Nagano, Japan [16], Vancouver, Canada [16], Uppsala, Sweden [16], Nancy, France [17], Szeged, Hungary [18], Nagpur, India [19], Berlin and Bavaria, Germany [20,21], and Nanjing, China [22]. Findings reveal distinctive near-surface ATs for each LCZ type under calm and clear weather conditions, which vary among cities with respect to their geolocation, size, surroundings, and relief [17,23,24]. Even though ATs are optimal for distinguishing thermal contrasts of LCZs [16,25], fixed sites are often insufficient in spatial representativeness, continuances, and variability of temperatures over a wide area [26]. Moreover, fixed sites can only account for a limited number of urban structure types and surface properties under the same climate background and weather conditions [22]. Mobile surveys, on the other hand, require much time and labor, are conducted above paved roads, and the quality and reliability of the observations are closely related to the path chosen [16]. Model simulations (e.g., UrbClim, MUKLIMO_3, Envi-met, and Weather Research and Forecasting model (WRF) [16,27,28,29]) have a solid foundation of physical theory, and provides the spatial distribution of canyon thermal conditions; nevertheless, representation of a realistic city is difficult, abundant parameters pose many challenges, and the reliability is highly dependent on model assumptions and accuracy [16].

With the advantages of providing practical spatiotemporal thermal information over a wide coverage area at a relatively low cost, remote sensing-derived land surface temperatures (LSTs) have been recently adopted in correlation to LCZs in cities such as Dubai, United Arab Emirates [30], Prague and Brno, Czech Republic [25], Yangtze River Delta and Pearl River Delta, China [31,32], and Phoenix and Las Vegas, USA [33]. The results demonstrate distinctive surface temperatures for different LCZs. The relationship between LSTs and ATs is very close and complicated in urban environments [3,34], and the LST characteristics of LCZs have certain differences from the AT characteristics [33,35,36], both spatially and temporally, which emphasizes the necessity of thermal investigation at varied spatial and time scales. However, due to the trade-off between spatial and temporal resolutions and the influence of cloud cover and heavy aerosols [37,38], previous studies that adopted high spatial resolution data (e.g., Landsat) have rarely examined multi-temporal, particularly diurnal, temperature effects (e.g., diurnal temperature range) of various urban forms and functions, whereas high temporal resolution data (e.g., Moderate Resolution Imaging Spectroradiometer (MODIS) [30,32]) were not frequently used because of the mismatch between their spatial resolution and LCZ maps, which limits the characterization of internal thermal variability of LCZs and signal interference from neighborhoods [32]. Meanwhile, many studies have utilized the remote sensing-based LCZ mapping approaches (typically the framework of the World Urban Database and Access Portal Tools (WUDAPT) that includes Landsat thermal bands for LCZ discrimination [39,40]) in correlation with the LSTs, which lose the independence between the LCZ classification and LST regimes [25,33].

The present study systematically investigates the LST characteristics across 18 different LCZ classes in the sprawling metropolis of Beijing, China, at annual, seasonal, monthly, and diurnal scales from July 2017 to June 2018. A geographic information system (GIS)-based method is used to classify LCZs based on five morphological and coverage indicators derived from a city street map (CSM) and Landsat images, and a spatiotemporal fusion model to generate hourly 100-m resolution LSTs by blending Landsat, MODIS, and FengYun (FY)-2F LSTs. This study is different from previous studies in that: (1) the methods cover as many LCZ types, spatial details, and time scales as possible and permit independence between the LCZ mapping and LST derivation, and thus unbiased investigation of the LCZ-LST correlation; and (2) the comprehensive impacts of urban morphology and surface cover are focused at multi-temporal scales. The aim of this study is to assess how different urban forms and functions are related to LST variations from a high spatiotemporal scale perspective [41] based on the LCZ classification and ultimately provide insight into the most effective urban form and design strategies for urban thermal regulation and sustainable planning.

## 2. Study Area and Data

### 2.1. Study Area

**Figure 1:**
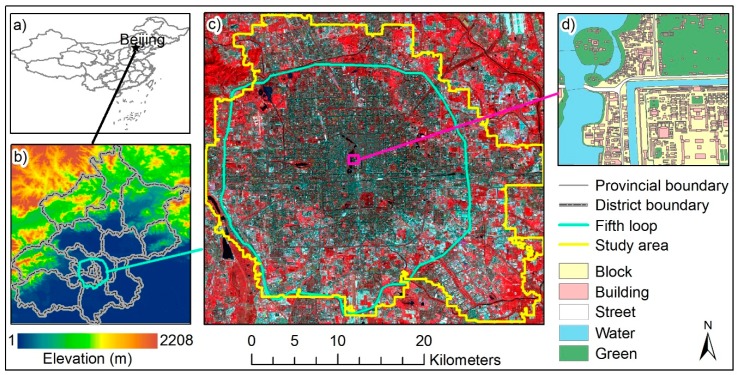
General view of the study area. (**a**) Administrative division of China. (**b**) Elevation of Beijing. (**c**) Landsat-8 pseudo color composite image of the study area on September 12, 2017. (**d**) City street map (CSM) of the subset area (**c**).

Beijing (39°28′–41°05′ N, 115°25′–117°30′ E) is the capital of China (Figure 1b), with a population of 22 million and built-up areas of 1401 km^2^ in 2016 (cited from the statistical yearbook of Beijing, 2016). The elevations range from 3 m in the southeastern plains to 2047 m in the northwestern mountains. With a temperate monsoon climate, the city experiences hot, wet summers and cold, dry winters [42], and with rapid urbanization, significant UHI events have been repeatedly reported [4,38,42,43].

Due to the limited access to morphological data of the entire Beijing area, only the central area within and around the 5th Ring Road of Beijing (Figure 1c), which has extensive human activities, was selected for use in this study. The area has experienced more than 3000 years of change and grown in concentric zones [4,44], with various forms and functions of buildings, including historic sites (e.g., the Forbidden city), old (e.g., Hutong) and new residential buildings, modern commercial centers, office buildings, industrial areas, transportation hubs, and allotments, located among roads, lakes, rivers, parks, and parking lots (Figure 1d), contributing to the analysis of various types of LCZs. The elevation change in the study area is small (19–89 m), ensuring a simple relief for the analysis of thermal characteristics of LCZs [13,16].

### 2.2. Data

The analyses of this study are based on a CSM, a land cover map, and multi-temporal Landsat, MODIS, and FY-2F images. For the sake of brevity, an overview of the data is listed in Table 1 and Table 2. The three-dimensional CSM (e.g., Figure 1d, obtained from the NAVINFO, http://www.navinfo.com/en/aboutus/index.aspx) provides crucial information on blocks, buildings, streets, water, and green spaces that were used to characterize the urban morphology. This was in a vector format which was then converted into a raster format with a spatial resolution of 15 m in this study to match with the Landsat-8 Operational Land Imager (OLI) data. The Landsat-8 OLI data (downloaded from the United States Geological Survey (USGS), http://earthexplorer.usgs.gov/) were mainly used to derive surface coverage indicators and the land cover map (downloaded from the Tsinghua University, http://data.ess.tsinghua.edu.cn/) was a supplement for distinguishing vegetation types (Section 3.1).

To analyze the thermal behavior of LCZs, LSTs were collected from Landsat-8 Thermal Infrared Sensor (TIRS), Terra and Aqua MODIS, and FY-2F Stretched Visible and Infrared Spin Scan Radiometer (SVISSR). The principles of data selection were as follows: (1) the LST observation time should be close to the CSM measurement time (i.e., 2016) so that the LSTs correspond to the LCZs derived from the CSM; (2) the Landsat and MODIS should form at least two pairs of clear reference images (cloud < 10%) in the study area within three months to preserve image similarity and derive conversion coefficients (Equation (1)); (3) the MODIS and FY-2F should form at least three pairs of clear reference images (cloud < 10%) in a diurnal cycle and the FY-2F should be clear for the rest of the day; and (4) the datasets should span at least one year covering as many months as possible for annual and monthly thermal analysis. Based on these principles, 10 Landsat, 122 MODIS, and 696 FY-2F scenes in 2017–2018 were chosen for use (Table 2).

Landsat-8 overpassed the study area at 10:54 every 16 days (the practical period of clear-sky images may be longer due to cloud coverage), and the LSTs were retrieved from band 10 at a spatial resolution of 100 m using a single-channel algorithm (Appendix B, errors < ±1.5 K when water vapor content <3 g/cm^2^) [45,46]. MODIS overpassed the study area four times a day (at ~11:00, ~13:30, ~22:00 and ~02:00), and the LSTs were retrieved from bands 31 and 32 at a spatial resolution of 1 km using a generalized split-window algorithm (errors < ±2.0 K in most cases) [47]. FY-2F provided hourly thermal observations (during 00:00–23:00) at a spatial resolution of 5 km, and the LSTs were retrieved using a split-window algorithm accounting for water vapor content (errors < ±2.0 K when water vapor content <3 g/cm^2^) [48].

All the above data were co-registered and subset to the study area, while low-quality pixels heavily affected by clouds, aerosols, and water vapor content were masked out.

## 3. Methods

**Figure 2:**
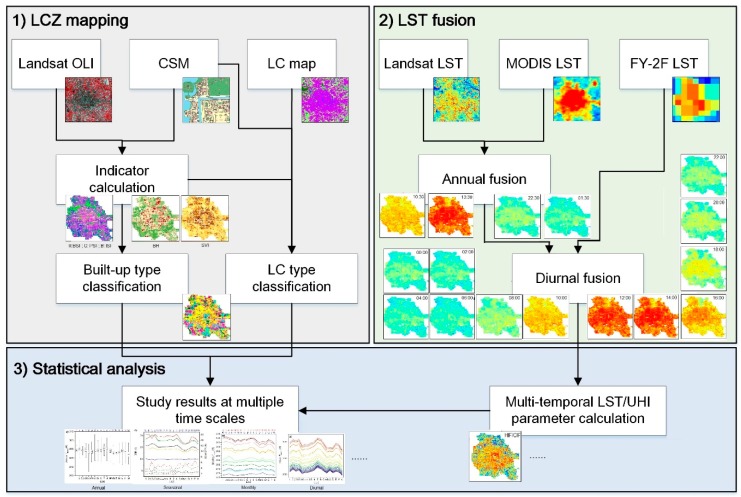
Conceptual workflow of this study.

The overall workflow of this study is shown in Figure 2. It consists of three major steps: (1) LCZ mapping, (2) LST fusion, and (3) statistical analysis. The first involves indicator calculation, built-up type classification (LCZs 1–10), and land cover type classification (LCZs A–G); and the second step implements the annual fusion of Landsat and MODIS LSTs, which were then followed by the diurnal fusion of Landsat-like and FY-2F LSTs to obtain hourly 100-m resolution LSTs. These two steps are completely different in data and method in order to maintain independence between LCZs and LSTs. In the final step, multi-temporal LST and UHI parameters were derived from the fused LSTs (step 2) and correlated to the LCZs (step 1) to discover the effects of urban forms and functions on LSTs and UHIs at varied time scales.

### 3.1. Local Climate Zone Mapping

**Figure A1:**
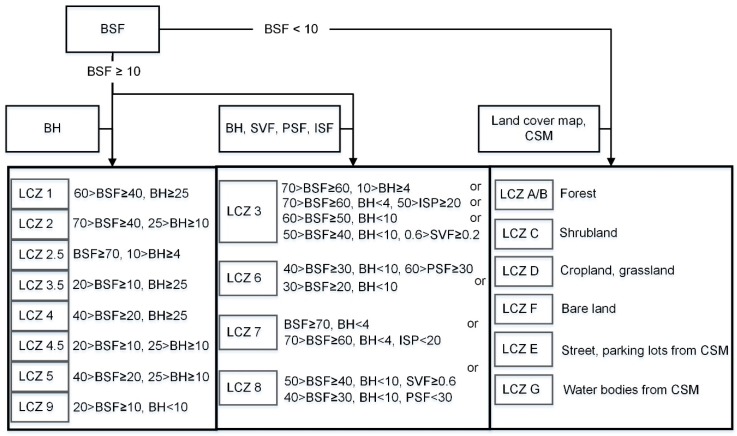
Flowchart of the LCZ mapping approach [35].

Different types of urban forms and functions were systematically classified into the LCZ scheme in this study, which was mapped using a GIS-based method developed by Quan [35] (flowchart in Figure A1). This method was chosen because it is completely independent from thermal information during the LCZ classification; it uses a clearly-defined decision-making algorithm requiring a small number of indicators that are easy to calculate, and it had been validated in the Beijing area using field samples (90% agreement) [35].

First, five morphological and coverage indicators defined by Stewart and Oke [13] were derived, including the building height (BH), building surface fraction (BSF), sky view factor (SVF), pervious surface fraction (PSF), and impervious surface fraction (ISF). BH and BSF were calculated based on the building attributes (i.e., height and footprint) in the CSM; SVF was derived using the Relief Visualization Toolbox based on the building height data added on the digital elevation model [35]; PSF was the sum of vegetation surface fraction (VSF), water surface fraction (WSF), and soil surface fraction (SSF) [13,35], which were calculated from the Landsat-8 red and near infrared bands [49], water layer of the CSM, and bare land class of the land cover map, respectively; and ISF was equal to 100%–BSF–PSF [13]. All the indicators were obtained at the pixel level and then individually aggregated in each block.

Second, blocks with BSF values > 10% were classified into best-matching built-up types by comparing the indicators with their typical ranges (Table A1 [13]) following a hierarchy (BH and BSF > SVF > PSF and ISF [35]). Some blocks were found to have BH and BSF exceeding the range of any standard LCZ [13]; therefore, built-up types of extremely compact low-rise (BH: 4–10 m, BSF: ≥ 70%, labeled as LCZ 2.5), extremely open high-rise (BH: ≥25 m, BSF: 10–20%, labeled as LCZ 3.5), and extremely open mid-rise (BH: 10–25 m, BSF: 10–20%, labeled as LCZ 4.5) were appended in this study (Table A1). First, seven LCZs were identified solely by BH and BSF, including LCZs 1–2 (compact high-/mid-rise), LCZs 3.5–5 (extremely open and open high-/mid-rise), and LCZ 9 (sparsely built). Then, five low-rise LCZs were determined by combining BH, BSF, and one of the remaining indicators (SVF, PSF, ISF), including LCZs 2.5–3 (extremely compact and compact low-rise) and LCZs 6–8 (open, lightweight and large low-rise). LCZ 10 (heavy industries) was not specified because the study area had eliminated most heavy industries, such as cement, steel, and coke before 2016, due to the City Master Plan for 2004−2020.

Finally, blocks with BSF values < 10% were categorized into certain land cover types (LCZs A–G) according to the land cover map and CSM. Specifically, due to the lack of tree geometry data, LCZ A (dense trees) and LCZ B (scattered trees) were less distinguishable, and therefore merged as one hybrid type: LCZ A/B, which corresponded to the forest class in the land cover map; shrublands, croplands and grasslands, and bare lands in the land cover map were categorized as LCZs C, D, and F, respectively; and streets and parking lots and water bodies from the CSM were used to determine LCZ E (bare rock or paved) and LCZ G (water), respectively.

### 3.2. Land Surface Temperature Fusion

**Figure A2:**
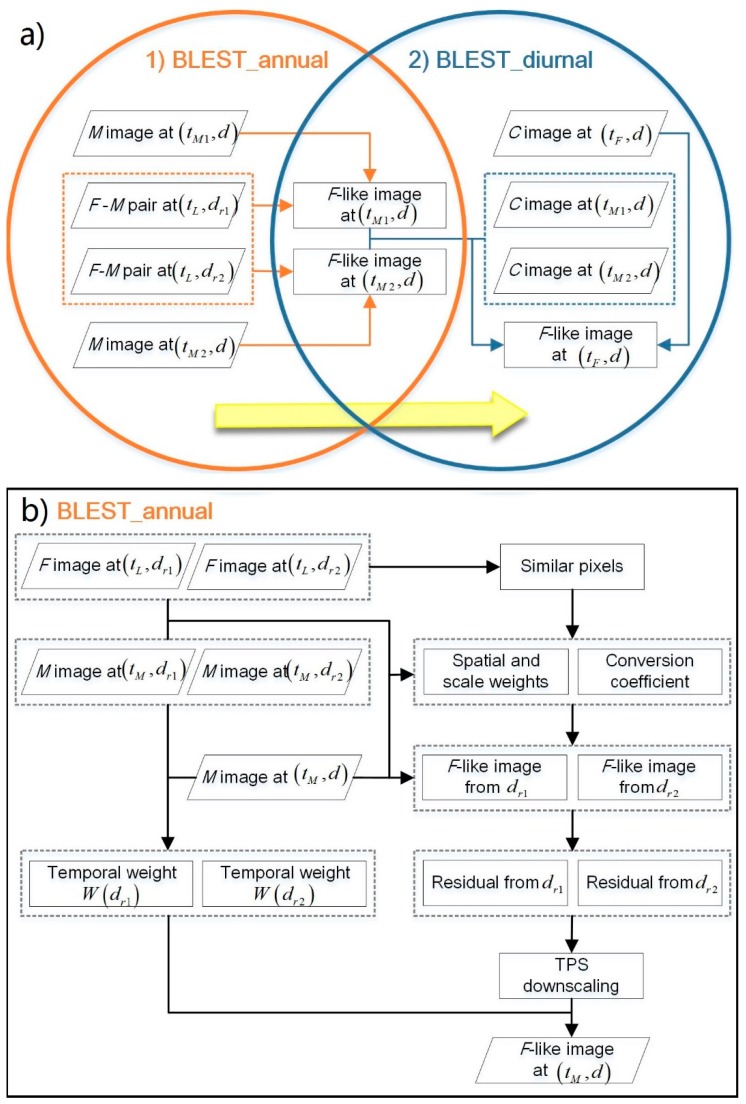
Flowchart of the BLEnding Spatiotemporal Temperature (BLEST) model [37]. (**a**) Two general steps: BLEST_annual and BLEST_diurnal; (**b**) the processes of BLEST_annual in details.

Hourly 100-m resolution LSTs were generated using a spatiotemporal fusion model termed BLEnding Spatiotemporal Temperatures (BLEST) developed by Quan et al. [37] (flowchart in Figure A2). BLEST was selected because it is suitable for heterogeneous landscapes by combining scale conversion coefficients and suitable for a long time span (e.g., one year) by accounting for land cover type changes through residual correction. The model had been evaluated in the Beijing area, demonstrating high performance (root mean square error (RMSE) < 1.0 K at the annual scale compared to Landsat images, RMSE < 1.0 K at the diurnal scale compared to MODIS images, and RMSE = ~2.5 K at the diurnal scale compared to in situ measurements [37]). BLEST involves two steps: BLEST_annual and BLEST_diurnal.

First, the Landsat and MODIS LSTs were blended at the annual scale to estimate Landsat-like LSTs at three or more MODIS observation times on the target day using the BLEST_annual approach (flowchart shown in Figure A2) [37]. The approach is based on the fact that the Landsat-like LST equals the Landsat LST observed on the reference day added to the LST change adjusted from the MODIS change and is weighted by all similar neighboring pixels, plus the downscaled residual (Equation (1)):

(1) $${\tilde{L}}_{d_{r}}\left(i,t_{M},d\right)=\underset{\mathrm{primary}\;\mathrm{estimate}\;\mathrm{at}\;\mathrm{an}\;\mathrm{annual}\;\mathrm{scale}}{\underset{︸}{\left[L\left(i,t_{L},d_{r}\right)+\sum_{s=1}^{S}w\left(s,t_{L},d_{r}\right)\times v\left(s,t_{L}\right)\times \left(M\left(i,t_{M},d\right)-M\left(i,t_{L},d_{r}\right)\right)\right]}}+R_{d_{r}}\left(i,t_{M},d\right)$$

where ${\tilde{L}}_{d_{r}}\left(i,t_{M},d\right)$ is the Landsat-like LST of pixel i at the MODIS observation time $t_{M}$ on the target day d estimated from the reference day $d_{r}$; L and M are the Landsat and MODIS LSTs, respectively; $t_{L}$ is the Landsat observation time; w is the weight or contribution of the thermally similar Landsat pixel s to the target pixel i in a neighborhood, determined by the spatial distance between pixel s and pixel i and the LST difference of pixel s between the Landsat and MODIS scales on $d_{r}$; $v\left(s,t_{L}\right)$ is the day-by-day conversion coefficient of the similar pixel s at $t_{L}$ between the Landsat and MODIS scales, calculated using the linear regression between the Landsat and MODIS LSTs on both reference days; R is the Landsat-scale residual downscaled from the MODIS-scale residual (i.e., the difference between the MODIS LSTs and the primary estimates averaged at the MODIS scale) using the thin plate spline algorithm.

To obtain higher accuracy, Landsat-like LSTs estimated from two reference days (${\tilde{L}}_{d_{r1}}\left(i,t_{M},d\right)$ and ${\tilde{L}}_{d_{r2}}\left(i,t_{M},d\right)$) were combined:

(2) $$\tilde{L}\left(i,t_{M},d\right)=W\left(d_{r1}\right)\times {\tilde{L}}_{d_{r1}}\left(i,t_{M},d\right)+W\left(d_{r2}\right)\times {\tilde{L}}_{d_{r2}}\left(i,t_{M},d\right)$$

where $d_{r1}$/$d_{r2}$ is the closet reference day before or after the target day; and $W\left(d_{r1}\right)$/$W\left(d_{r2}\right)$ is the temporal weight of the Landsat-like estimate from $d_{r1}$/$d_{r2}$, determined by the MODIS LST change from the reference day to the target day.

Second, under the similar framework to the BLEST_annual (i.e., similar pixel weighting, residual downscaling, and temporal weighting), hourly Landsat-like LSTs on the target day ($\tilde{L}\left(i,t_{F},d\right)$) were estimated by blending the former Landsat-like LSTs ($\tilde{L}\left(i,t_{M},d\right)$) and the FY-2F LSTs (F) using the BLEST_diurnal approach [37], as shown in Equations (3) and (4):

(3) $$\tilde{L}\left(i,t_{F},d\right)=W\left(t_{M1}\right)\times {\tilde{L}}_{t_{M1}}\left(i,t_{F},d\right)+W\left(t_{M2}\right)\times {\tilde{L}}_{t_{M2}}\left(i,t_{F},d\right)$$

with

(4) $${\tilde{L}}_{t_{M}}\left(i,t_{F},d\right)=\underset{\mathrm{primary}\;\mathrm{estimate}\;\mathrm{at}\;a\;\mathrm{diurnal}\;\mathrm{scale}}{\underset{︸}{\left[\tilde{L}\left(i,t_{M},d\right)+\sum_{s=1}^{S}w\left(s,t_{M},d\right)\times v\left(s,d\right)\times \left(F\left(i,t_{F},d\right)-F\left(i,t_{M},d\right)\right)\right]}}+R_{t_{M}}\left(i,t_{F},d\right)$$

where $t_{F}$ is the FY-2F observation time; ${\tilde{L}}_{t_{M1}}\left(i,t_{F},d\right)$ and ${\tilde{L}}_{t_{M2}}\left(i,t_{F},d\right)$ are the Landsat-like LSTs at $t_{F}$ estimated from two reference times ($t_{M1}$ and $t_{M2}$) in Equation (4), respectively, and $W\left(t_{M1}\right)$ and $W\left(t_{M1}\right)$ are their temporal weights, respectively; and v(s,d) is the diurnal conversion coefficient of pixel s on d between the Landsat and FY-2F scales, derived from the linear regression between the Landsat-like and FY-2F LSTs on both reference times. The calculation of weight w and residual R is similar to Equation (1) but at a diurnal scale between the Landsat-like and FY-2F images. More details on the selection of similar pixels and calculation of day-by-day and diurnal conversion coefficients, weights, and residuals can be found in a previous study [37].

### 3.3. Statistical Analysis

To quantitatively determine the thermal responses of LCZs at multiple time scales, parameters that represent the annual, seasonal, monthly, and diurnal LST dynamics were derived from the fused Landsat-like LSTs, including the annual, monthly, daily, daytime, nighttime, or hourly mean temperatures ($T_{\mathrm{mean}}$), annual temperature range (ATR), daily maximum or minimum temperature ($T_{\max}$/$T_{\min}$) and its time ($t_{\max}$/$t_{\min}$), and diurnal temperature range (DTR) (Table 3). They were calculated at each pixel and then overlaid with the LCZs, and their averages and standard deviations (SDs) or box plots were demonstrated for each LCZ to indicate the inter- and intra-class differences, respectively. One-way analysis of variance (ANOVA) was conducted on each LCZ pair for all the parameters to evaluate the statistical significance of inter-LCZ temperature differences, based on the non-parametric Kruskal-Wallis test [20,21,50], which was chosen over the parametric tests (e.g., *t*-test) because of its loose requirement on the normal distribution of the tested data (Section 4.1) [21,50].

Furthermore, the heat island or cool island (HI or CI) intensity (HII or CII) was calculated as the positive or negative LST difference of one LCZ type from the reference zone (i.e., LCZ D) and categorized into multiple levels: extremely strong (|HII/CII| > 6.5 K), strong (4.5 < |HII/CII| ≤ 6.5 K), moderate (2.5 < |HII/CII| ≤ 4.5 K), weak (0.5 < |HII/CII| ≤ 2.5 K), and neutral (|HII/CII| ≤ 0.5 K). Then, the HI or CI frequency (HIF or CIF, Table 3) was determined as the rate at which a certain HII or CII level occurs during the year, season, daytime, or nighttime. The HIF and CIF are comprehensive indices used to measure how much and for how long the LST differences between LCZs occur [22].

## 4. Results

### 4.1. A General View of the Local Climate Zones and Land Surface Temperatures

**Figure 3:**
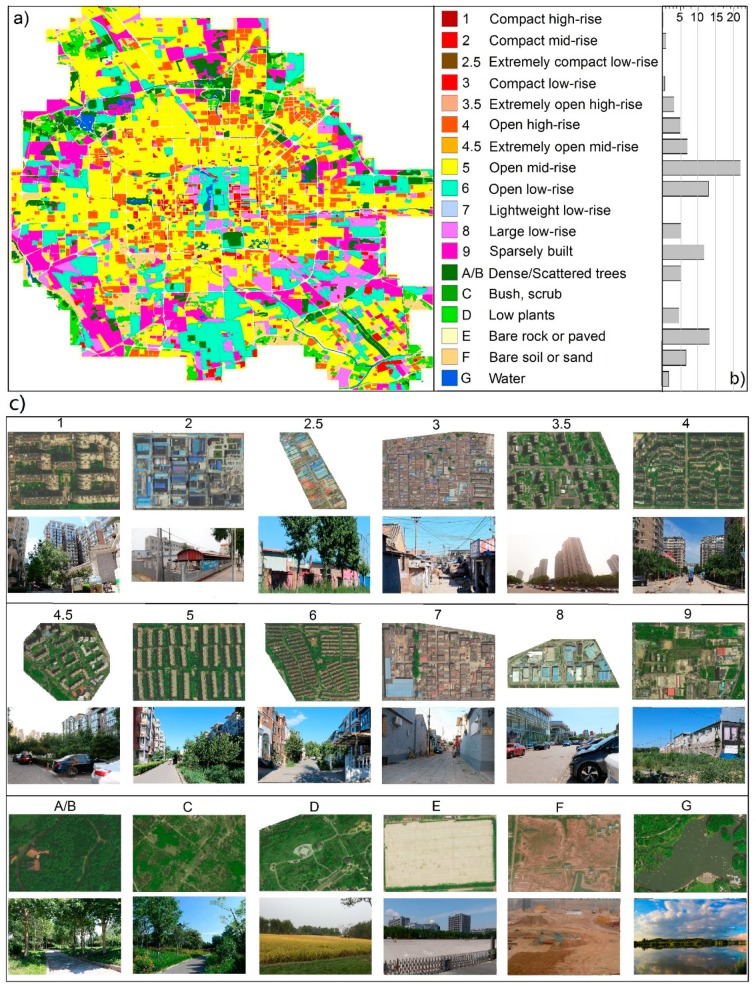
Local climate zones (LCZs) of the study area. (**a**) LCZ map; (**b**) percentage of each LCZ (%); (**c**) Google Earth images and field photos of examples of LCZs [31].

An LCZ map was generated over the study area (Figure 3), showing diverse types of urban forms and functions (Figure 3c) with different distribution patterns. The most frequent types are LCZ 5 (open mid-rise, 28.7%) and LCZ 6 (open low-rise, 12.8%), while the high-rise zones (LCZs 1, 3.5, and 4) account for 8.4%. The study area, in general, exhibits an annular pattern of building height: low-rise zones in the center (mainly historical buildings such as Hutongs and the Forbidden City), mid and high-rise zones in the middle, and low-rise zones in the periphery, which is in accordance with the concentric growth pattern of Beijing [44]. The compact built-up zones (LCZs 1–3, 12.0%) surround the center of the city (within the third loop), while LCZ 9 (sparsely built, mainly warehouses and car, furniture, or agricultural product markets) is typical of the outer part. The land cover types (LCZs A–D (9.9%), F (6.6%), and G (1.8%)) are mainly located in the parks (e.g., the Olympic forest park), construction sites, and lakes and rivers (e.g., the Kunming Lake and Hucheng River), scattered around the study area. Over 10% of the area is classified as three supplementary classes (LCZs 2.5, 3.5, and 4.5) due to a significant deviation in BSF from the standard definition in LCZs 3–5. Their LST differences from the standard ones were further explored. More details on the LCZ map can be found in a previous study [35].

**Figure 4:**
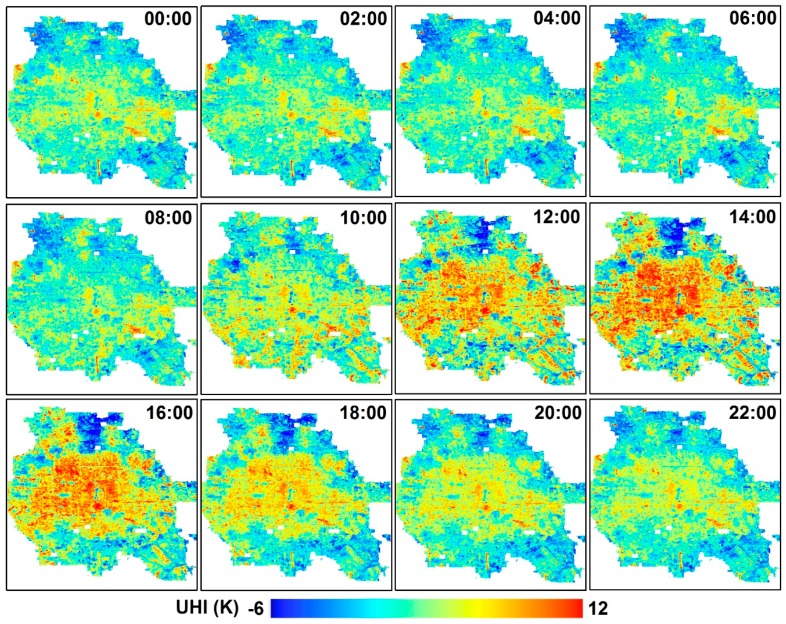
Urban heat islands (UHIs), i.e., land surface temperature (LST) differences from the reference zone (i.e., LCZ D), on September 7, 2017, demonstrated at an interval of two hours due to the page limit.

Hourly LSTs were generated at the 100-m spatial resolution on 29 days (Table 2) over the study area. Figure 4 shows the spatial distributions of UHIs (i.e., LST differences from the reference zone (i.e., LCZ D)) on September 7, 2017, as an example, revealing that the spatial details and diurnal dynamics of LSTs and UHIs were successfully reconstructed. The outer part of the study area was generally cooler than the inside, and hotspots were often associated with dense built-up areas. The road network and water boundaries were sharply demonstrated with deviated LSTs from the surroundings, particularly during 10:00–16:00, which can hardly be detected by MODIS, FY-2F, or other moderate or low spatial resolution images. The large forest parks exhibited lower LSTs than the built-up areas during the entire diurnal cycle in September, while their spatial patterns gradually changed with the hour, suggesting variations during the day and night.

**Figure 5:**
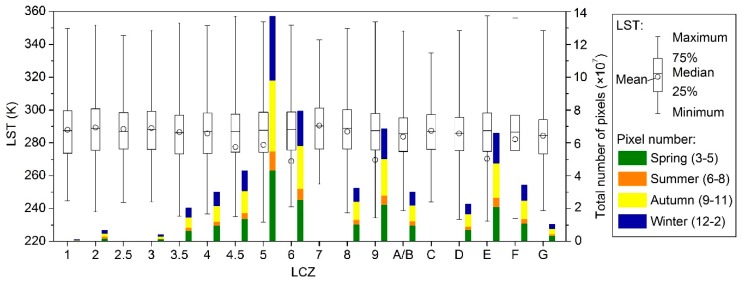
Statistics of all usable LST values for each LCZ across the study area and period. Spring: March–May; Summer: June–August; Autumn: September–November; Winter: December–February.

A certain degree of spatial correspondence was observed between the LCZs and LSTs according to Figure 3 and Figure 4. Figure 5 shows the amounts and distributions of all usable LST values for each LCZ across the study area and period. In general, the amounts of LSTs of different LCZs followed the same pattern as their portions in the study area (Figure 3b). For each LCZ, most LSTs were evenly distributed in spring, autumn, and winter, while a relatively small amount was found in summer when a large amount of rain and clouds formed in the Beijing urban area to contaminate the satellite thermal observations [4], which could not be reconstructed by the fusion method in this study. This resulted in left-skewed distributions of LSTs with lower means with respect to the medians, particularly for the LCZs with larger summer-winter differences in the total amounts, such as LCZs 4.5, 5, 6, 9, and E. Thus, methods such as the *t*-test that require normally distributed data were considered not suitable to test the significance level in this study; instead, the non-parametric Kruskal-Wallis test [50] was used (Section 4.3). Moreover, no clear-sky LST images were available for the entire month of August (Table 2), which led to the underestimation of the annual average considering higher LSTs in August [38]. Nevertheless, this impact would be greatly reduced during the difference analysis within or among LCZs. The details on the association between LSTs and LCZs at multi-temporal scales are demonstrated in Section 4.2, Section 4.3 and Section 4.4.

### 4.2. Annual and Monthly Land Surface Temperature Variations

**Figure 6:**
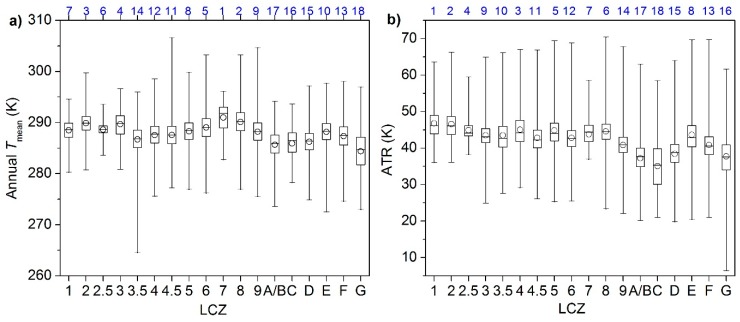
Statistics of the (**a**) annual mean temperature (annual $T_{\mathrm{mean}}$) and (**b**) annual temperature range (ATR) for each LCZ in the study area. The blue number on the top axis expresses the sequence of each LCZ from high to low in terms of the average. Please see Figure 5 for the explanation of the boxes.

**Figure 7:**
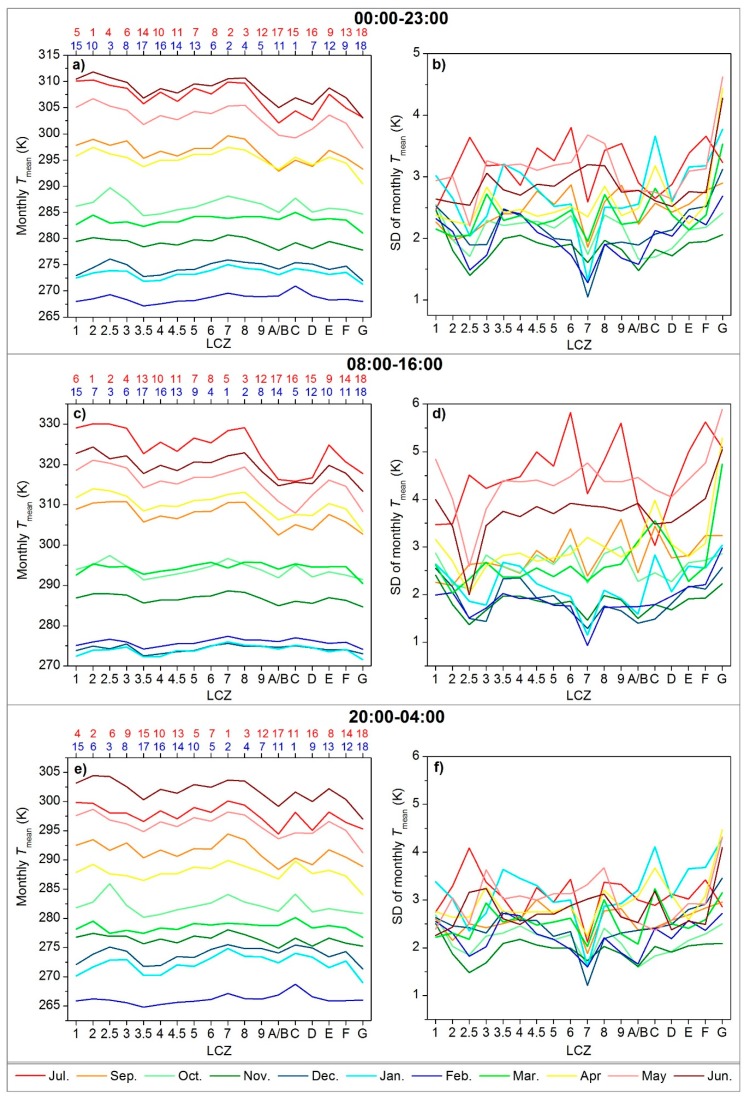
Monthly mean temperature (monthly $T_{\mathrm{mean}}$) and its standard deviation (SD) for each LCZ in the study area, calculated at (**a**,**b**) all hours (00:00–23:00), (**c**,**d**) daytime (08:00–16:00), and (**e**,**f**) nighttime (20: 00–04:00). The red and blue numbers on the top axes of (**a**,**c**,**e**) express the sequence of each LCZ from high-to-low in terms of monthly $T_{\mathrm{mean}}$ during April–September and October–March, respectively.

The annual temperature parameters (i.e., annual $T_{\mathrm{mean}}$ and ATR) and monthly temperature parameters (i.e., monthly $T_{\mathrm{mean}}$ (00:00–23:00), monthly daytime $T_{\mathrm{mean}}$ (08:00–16:00), and monthly nighttime $T_{\mathrm{mean}}$ (20:00–04:00)) were plotted for different LCZs in Figure 6 and Figure 7. As the LSTs of different hours, days, and months were also affected by dynamic factors such as synoptic situations [25], the rank order of 18 LCZs was derived for each parameter according to the mean values from high-to-low to indicate the inter-LCZ differences at multi-temporal scales. Meanwhile, to evaluate the intra-LCZ variability, the SDs of parameters for different LCZs were compared with each other.

Relatively large intra-LCZ variability was observed at the annual scale (Figure 6) considering both spatial variations and temporal dynamics with high resolution. The smallest variations in the annual $T_{\mathrm{mean}}$ were associated with LCZs 1–3 (compact high, mid, or low-rise and extremely compact low-rise), LCZ 7 (lightweight low-rise), and LCZ C (bush, scrub) because of their small numbers of observations and blocks compared to others (Figure 5). On average, annual $T_{\mathrm{mean}}$ and ATR demonstrated significant differences among LCZs (up to 6.6 K for annual $T_{\mathrm{mean}}$ and 11.6 K for ATR; Figure 6) with higher values in the built-up types and lower ones in the land cover types, which confirms the finding of [51] that the annual mean and amplitude of LSTs can be used to distinguish diverse LCZs. The highest annual $T_{\mathrm{mean}}$ was obtained for compact and mid- and low-rise building types with little vegetation, including LCZ 7 (lightweight low-rise), LCZ 8 (large low-rise), LCZ 2 (compact mid-rise), and LCZ 3 (compact low-rise) (in descending order), due to small SVF, high impermeability, and little vegetation, while the lowest annual $T_{\mathrm{mean}}$ was associated with water and vegetated zones, including LCZ G (water), LCZ A/B (dense or scattered trees), LCZ C (bush, scrub), and LCZ D (low plants) (in ascending order), because extensive pervious surfaces lower surface temperatures. The remaining LCZs exhibited an annual $T_{\mathrm{mean}}$ of 286.7–289.0 K. Among the built-up types, LCZ 3.5 (extremely open high-rise) and LCZ 4 (open high-rise) had the lowest annual $T_{\mathrm{mean}}$, whereas among the land cover types, LCZ E (bare rock or paved) had the highest annual $T_{\mathrm{mean}}$, even higher than some built-up types (LCZ 3.5–4.5), primarily caused by the low infiltration rate [33].

Similar to the annual $T_{\mathrm{mean}}$, the lowest ATR emerged for the water and vegetated surface types (LCZs A–D and G) and LCZ E had the highest ATR among the land cover types (Figure 6b). However, different from the annual $T_{\mathrm{mean}}$, the highest ATR emerged for the compact and high- and mid-rise building types, including LCZ 1 (compact high-rise) and LCZ 2 (compact mid-rise), whereas LCZ 9 (sparsely built) had the lowest ATR among the built-up types because seasonal variations in abundant vegetation covers greatly decrease LSTs through evapotranspiration and shades in the growing season but slightly decrease or even increase LSTs during the defoliation period [22]. High-rise built-ups (LCZs 1 and 4) were found to have lower annual $T_{\mathrm{mean}}$ (order = 7 and 12) but higher ATR (order = 1 and 3), probably owing to their thermal sensitivity to the seasonal changes in solar radiation angles and intensities through shade effects and total surface gain.

Figure 7 demonstrates the monthly LSTs—the highest in June and July (excluding the August) and the lowest in February. Strong inter-LCZ differences were observed during April–September (6.7–9.4 K), with a similar pattern with the annual $T_{\mathrm{mean}}$, where LCZ 9 had a lower order (order = 12, indicating lower monthly $T_{\mathrm{mean}}$) than that at the annual scale (order = 9), further revealing similar seasonal variations to those in ATR. The other months, however, demonstrated weaker differences among LCZs (3.0–5.3 K), because reduced solar radiation and vegetation cover weaken the differences in warming and cooling rates among LCZs. The sequence of the LCZs also changed—LCZs A/B, C, and D showed an increase in LSTs with respect to the built-up types due to the leaf abscission and crop harvesting, which denote more thermal response from bare soils, contributing to the cool island generation (Section 4.4). Meanwhile, LCZs 1 and 4 had a significant drop in the sequence (order = 15 and 16) during October–March, corresponding to their larger ATR.

The intra-LCZ variability is mainly attributed to the differences in location, surrounding, size, layout, physical or functional properties (e.g., soil moisture, subclasses), surface relief, anthropogenic heat (e.g., traffic load), and micro-climate of the LCZ units [16,18,27] in the wide area. As the impacts of the above factors are generally enhanced with stronger insolation and disturbance during the summer [17], larger heterogeneity was observed within the same LCZ type during summer months (Figure 6b,d,f). The errors in LCZ mapping and LST fusion also contributed to the intra-LCZ differences to some extent, which would be more pronounced in winter because the LCZ map was built upon the summertime vegetation, water, and soil information [35], and the regression of the diurnal conversion coefficients (v in Equation (4) for LST fusion) was less stable during the winter due to low diurnal temperature variations [37]. Therefore, the impacts of the model errors may not be as significant as those of other factors in this study. Otherwise, irregular patterns of SDs were observed among LCZs between different months.

### 4.3. Diurnal Land Surface Temperature Variations

**Figure 8:**
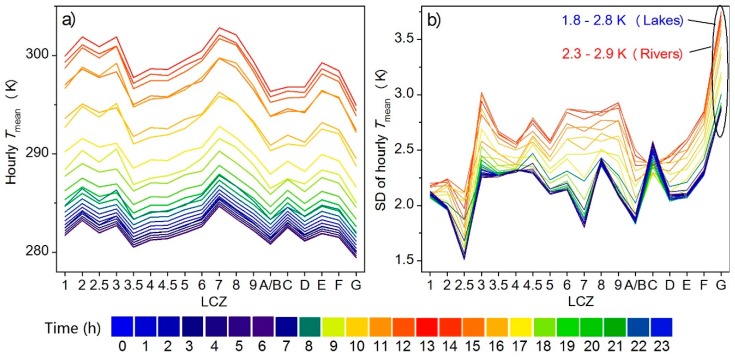
Hourly mean LST (hourly $T_{\mathrm{mean}}$, (**a**)) averaged for one year and its SD (**b**) for each LCZ in the study area. The blue and red texts on the upper right corner (**b**) express the SD of the LCZ G when only lakes and rivers were accounted for. The box plot for each hour and each LCZ was not demonstrated due to the page limit.

Hourly $T_{\mathrm{mean}}$ values were averaged for one year and plotted in Figure 8a, which shows a typical diurnal cycle with the highest LSTs around 13:00 and the lowest LSTs around sunrise. The inter-LCZ differences in each hour generally followed the same pattern as the annual $T_{\mathrm{mean}}$ (Figure 6a)—greater temperature contrasts corresponding to larger deviations in surface structure and cover [22]; higher $T_{\mathrm{mean}}$ for compact zones than open or sparse zones; larger compact-open differences for mid-rise buildings; and increases in LST with decreases in building height (except for the compact low-rise that was warmer during the day but cooler at night than the compact mid-rise). Moreover, both the inter- and intra-LCZ differences (Figure 8a,b) demonstrated diurnal patterns with higher intensities during the day (5.2 (06:00)–7.9 (13:00) K) and lower intensities at night (5.2 (05:00)–5.4 (19:00) K). Figure 8b shows that LCZ G (water) had the largest intra-class variations (SDs = 2.9–3.8 K). LCZ G mainly includes lakes and rivers, located all around the study area and surrounded by forests, low plants, bare soil, buildings, or roads (Figure 3). After separating the lakes and rivers, the SDs of LCZ G became 1.8–2.8 K (only for lakes) and 2.3–2.9 K (only for rivers), where the mean LSTs of the lakes were 4.0 (midnight)–6.0 K (noon) lower than those of the rivers. Diversities in the water depth, width and rate of flow can also influence the water temperature variability [25]. This further underlines the importance of exploring intra-LCZ differences and the necessity of local adjustment when adopting the standard LCZ scheme.

**Figure 9:**
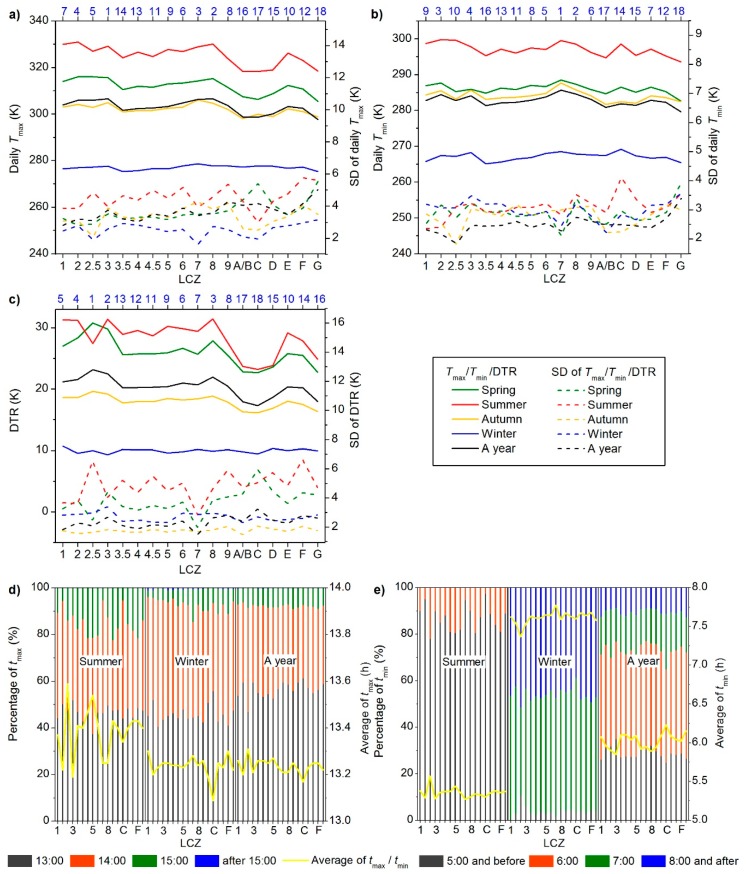
Means and SDs of diurnal parameters for each LCZ averaged across one year or season in the study area. (**a**) Daily maximum LST (daily $T_{\max}$); (**b**) daily minimum LST (daily $T_{\min}$); (**c**) diurnal temperature range (DTR); (**d**) percentage of the time period of daily $T_{\max}$ ($t_{\max}$); (**e**) percentage of the time period of daily $T_{\min}$ ($t_{\min}$). The blue numbers on the top axes (**a**–**c**) express the sequence of each LCZ from high to low in terms of the averages in one year.

Diurnal parameters of each LCZ in one year and four seasons are demonstrated in Figure 9. According to the annual averages, the ranking of the daily $T_{\max}$ and $T_{\min}$ was generally consistent with the inter-LCZ pattern of annual $T_{\mathrm{mean}}$ but with weaker inter-LCZ variability at night, leading to higher or lower DTRs for LCZs with higher or lower $T_{\max}$; i.e., LCZs 2.5, 3, and 8 had the highest DTRs, while LCZs G and A/B had the lowest, and LCZs E and F had the highest DTRs among the land cover zones, which is consistent with previous reports on annual DTR differences between built-up and vegetated or water surfaces [52]. Each LCZ showed the largest DTR in summer and the lowest in winter, where LCZs A–D had a weaker seasonal variability in DTR than the built-up zones, mainly due to the phenological changes. Both the daily $T_{\max}$ and DTR demonstrated weaker differences among LCZs in winter, while the daily $T_{\min}$ registered significant inter-LCZ differences in winter than the daily $T_{\max}$ because the collective heating system of Beijing in winter increases the anthropogenic heat output, which has a larger impact on LSTs at night [4,10]. The intra-LCZ variations in daily $T_{\max}$ and DTR were stronger than those in daily $T_{\min}$, consistent with the pattern in the one-year averaged hourly $T_{\mathrm{mean}}$ (Figure 8b).

**Figure 10:**
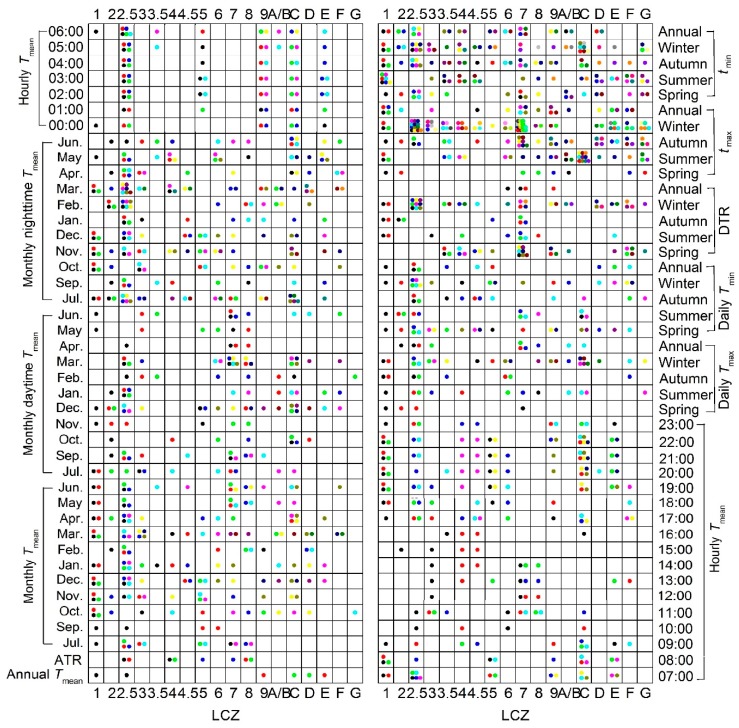
Results of Kruskal-Wallis tests for annual, seasonal, monthly, and diurnal parameters between all LCZ pairs. For each parameter, each dot indicates that the corresponding LCZ is insignificantly different (*p* > 0.05) from another LCZ represented with the dot of the same color. The number of dots in each square indicates the times of the LCZ registering with insignificance for a certain parameter.

Figure 9d shows that 45–50% of the pixels had $t_{\max}$ as 13:00 for each LCZ in summer and winter, while 30–45% reached the daily $T_{\max}$ at 14:00 in summer and 40–55% in winter. For each LCZ, the average of $t_{\max}$ was earlier in summer than in winter. Moreover, winter had the latest time to start warming up in the morning, i.e., mostly 5:00 or earlier in summer, ~6:00 in spring and autumn, and mostly 7:00 or later in winter (Figure 9e), which corresponded to the time of sunrise in the annual cycle. The difference among LCZs in the average of $t_{\max}$/$t_{\min}$ was within 0.5 h (<1 h for $t_{\max}$ in summer), which was shorter than the time interval of the fused Landsat-like LSTs, and thus, considered insignificant (Figure 10).

The Kruskal-Wallis test was conducted against each parameter between all LCZ pairs to explore the significance of inter-LCZ differences. Each pair of “dots” in the same color in Figure 10, for a certain parameter (i.e., in the same line), demonstrates an insignificant LST difference (*p* > 0.05) between the corresponding pair of LCZs. In general, LCZ pairs with significant differences (*p* < 0.05) prevailed (95%), and none of the LCZs continued to show insignificant differences with any other LCZ for varied parameters and time periods, suggesting that the LCZ scheme and the proposed method were appropriate to delineate the multi-temporal LST characteristics.

The largest number of “dots” (insignificant differences) was registered for LCZ 2.5 (extremely compact low-rise), followed by LCZ C (bush, scrub), LCZ 7 (lightweight low-rise), and LCZ 1 (compact high-rise). This was mostly attributed to their relatively weak LST differences and small numbers of pixels or blocks (i.e., small sample size) in the study area (<0.2%, Figure 3) [20]. Specifically, LCZs 1 and 2.5 had many small blocks (<100 m^2^), whose LST signals were likely mixed with the surrounding areas. Considering that the existing LCZ scheme defines the zones spanning hundreds of meters to several kilometers [13], some local adjustment is required to obtain a more appropriate description of these blocks. In contrast, LCZ G (water) was the most distinguishable one, followed by LCZ A/B (dense or scattered trees), LCZ 2 (compact mid-rise), and LCZ 3.5 (extremely open high-rise). This was strongly supported by their large degree of LST differences from the other LCZs.

Second, the LCZ 7-LCZ 8 pair was registered the most with insignificant differences—20 out of 84 parameters were insignificant (*p* > 0.05), most likely due to their similar thermal responses (both very high, e.g., Figure 6a, induced by little vegetation, prevailed impervious or soil surfaces with similar building heights) and the limitation of the current method in correctly separating them by material and building size [35]. On the contrary, the LCZs with different building heights but similar compactness and surface covers (e.g., LCZs 1–3 or LCZs 4–6) showed more distinctive LST characteristics, indicating that the building height and canyon structure have a decisive influence on the thermal behavior. In comparison to the instantaneous nadir or near nadir observations (e.g., Landsat) that weaken the impact of vertical surfaces, especially for dense buildings [25], the characterization of annual and diurnal LST dynamics in this study (embodied in the BLEST model and thermal parameter calculation) contributed to reflecting temporal changes in shaded areas (referring to the building height and direct solar radiation) for varied solar radiation angles during the year. The characterization also helped to suppress the impact of thermal anisotropy [53,54], considering that multiple view positions (MODIS) were fused in BLEST and parameters were averaged at different time scales [25].

Finally, more insignificant differences among LCZs were observed during the winter (104 pairs) and nighttime (78 pairs) than during the summer (62 pairs) and daytime (52 pairs). The annual parameters (e.g., annual $T_{\mathrm{mean}}$, ATR) were better differentiated among various LCZs than the diurnal parameters, confirming the role of the annual parameters in classifying LCZs [39,51] but suggesting less effective diurnal parameters. There was, however, one exception—the annually averaged DTR referred to both the diurnal thermal properties and annual cycle information and had only two LCZ pairs with insignificant differences. Therefore, the annually averaged DTR (some may also consider related factors such as annually averaged thermal inertia) could be a good candidate contributing to the LCZ classification.

### 4.4. Frequencies of Heat Islands and Cool Islands at Multi-Temporal Scales

**Figure 11:**
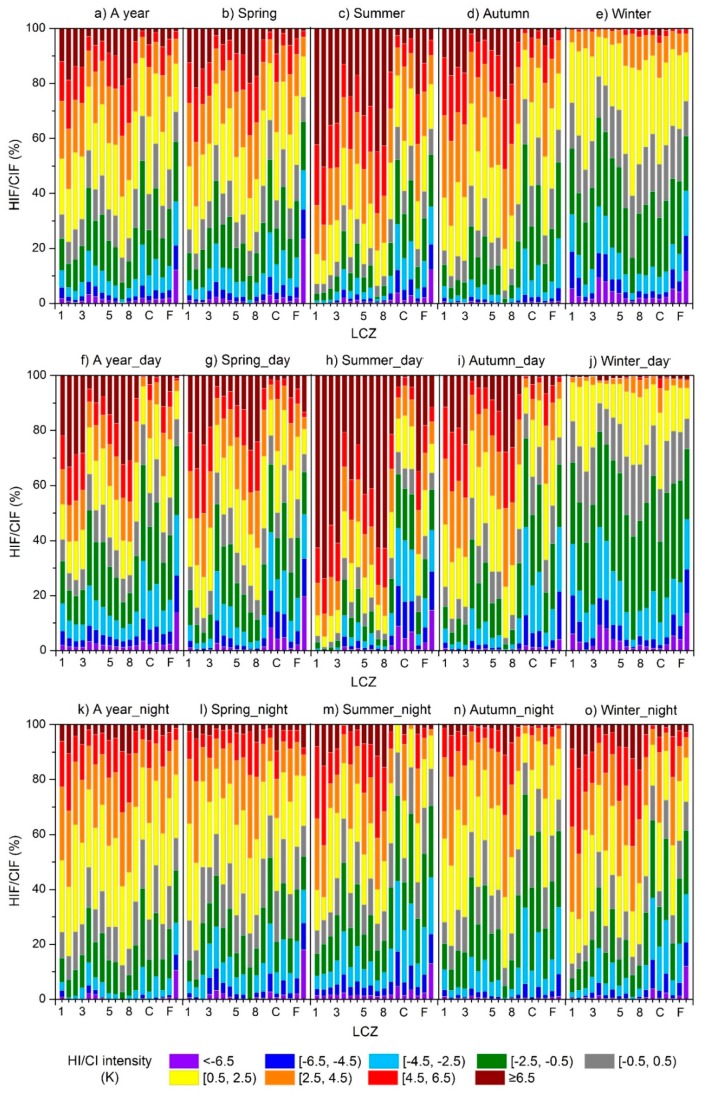
Frequencies of heat and cool islands (HIF and CIFs) at different intensities for each LCZ, calculated over different time periods: (**a**) One year; (**b**) spring; (**c**) summer; (**d**) autumn; (**e**) winter; (**f**–**j**) one year and four seasons during the daytime (08:00–16:00); (**k**–**o**) one year and four seasons during the nighttime (20:00–04:00).

The HIFs and CIFs of different LCZs were calculated using LCZ D as a reference (Figure 11) to provide a more comprehensive analysis of the intensity and frequency of the inter-LCZ differences (represented as HIs and CIs). In general (Figure 11a–e), built-up zones in the study area presented more hours of HIs (44.4–83.4%) than CIs (7.6–43.0%) in a year, where compact built-up zones formed more frequent HIs (67.7–77.7%) than open built-up zones (44.4–68.0%). Specifically, LCZs 2, 7, and 8 had about 80% of the time in a year generating weak (0.5–2.5 K)–extremely strong (≥6.5 K) HIIs, forming the most intense local HIs. On the contrary, LCZs G and A/B formed local CIs (< -0.5 K) for over half of the time (58.8% and 51.9%) and indicated stable cooling effects; LCZ G generated intense CIs (< -4.5 K) for 21.1% of the time. LCZ 4 showed the most similar HIF and CIF pattern to LCZ D; i.e., 40–45% of the time, LCZ 4 generated His and CIs, and 15% of the time, it was neutral.

Seasonal and diurnal variations were also observed. In summer (Figure 11c), HIs dominated the most LCZs (except for LCZs A/B, D, and G) from 59.1% (LCZ F) to 93.7% (LCZ 2) of the total hours (5.5% to 50.4% at extremely high intensity, i.e., >6.5 K). LCZ 3.5 (extremely open high rise) recorded the lowest HIFs (64.3%) among the built-up types, followed by LCZ 9 (sparsely built) at 69.4%. Surprisingly, LCZs A/B and G were warmer than LCZ D for 35.8% and 36.5% of the total hours (mostly at night). This was also reported in some of the previous studies based on the AT measurements and explained by their large heat capacity that decreases the cooling rate of the close atmosphere at night [22]. Comparatively, CIFs increased greatly in winter (Figure 11e) mostly during the day (Figure 11j) for all the built-up zones (39.2–79.5%), primarily due to the reduced solar radiation gain in winter and seasonal changes in LCZ D [55,56], e.g., the surface coverage, morphology, and moisture that enhance its warming and cooling rate in winter [57] (also seen in Figure 9c that the DTRs of most built-up zones were lower than that of LCZ D in winter [52]). The total hours of CIs were longer for open and high-rise built-up zones but shorter for compact and low-rise built-up zones. However, with specific types, daytime CIs have very complex mechanisms that are yet to be fully understood [22,58]. HIs were rather weak (0.5–2.5 K) in winter and 12.7–22.6% of the hours had neutral events (-0.5–0.5 K). This seasonal pattern compared well with the previous studies that documented strong HIs in summer and CIs in winter in Beijing [56,57,59]. The sequence of the HIFs or CIFs for different LCZs during the day or night was similar to that at the annual scale. However, larger discrepancies among LCZs were recorded during the day, especially for the extremely intense HIs (>6.5 K), i.e., 4.9 (LCZ 4)–33.2% (LCZ 2), which were more than twice that at night (1.9–14.8%).

## 5. Discussion

### 5.1. Relationship between Land Surface Temperatures and Morphological and Coverage Indicators

To discover driving mechanisms underlying LST and UHI differences among LCZs, Spearman’s rank correlation analysis was conducted between ten primary thermal parameters (i.e., annual $T_{\mathrm{mean}}$, ATR, summer or winter and daytime or nighttime $T_{\mathrm{mean}}$, summer or winter DTR, and annual HIF or CIF, Section 3.3) and five key morphological and coverage indicators of LCZs (i.e., SVF, BH, BSF, VSF, and ISF, Section 3.1), where water bodies were excluded to facilitate unbiased correlation [4,60,61]. Table 4 shows the Spearman’s rank correlation coefficient (ρ) for each LST-indicator pair, which was selected over widely used Pearson’s correlation because it is a non-parametric measure with no assumptions on the data frequency distribution [60,62].

The relationship between LST parameters and LCZ indicators significantly varies with time (Table 4). For summer parameters (i.e., summer daytime or nighttime $T_{\mathrm{mean}}$ and summer DTR), VSF yields the highest negative correlations (−0.49 ≤ ρ ≤ −0.35) among the five indicators, which is resulted from the well-established mechanism that the vegetation increases latent heat fluxes via evapotranspiration and casts shadows via tree canyons, generating a cooling effect especially during the daytime with respect to the nighttime when the evapotranspiration and shades disappeared [5,9,10,11,60]. However, due to the vegetation phenology and seasonal insolation variation (e.g., intensity and angle), the vegetation cooling effect is greatly reduced and even inversed (i.e., positive correlation due to the increased bare soil contribution) in cold (leaf-off) winter (0.02 ≤ ρ ≤ 0.21) [9,10]. Hence, it is reasonable to find that smaller ATR and annual $T_{\mathrm{mean}}$ correspond to a larger VSF. This mainly explains the seasonal thermal differences between the vegetated types (LCZs A–D) and built-up types (LCZs 1–9), as shown in Figure 6, Figure 7 and Figure 9.

For winter parameters (i.e., winter daytime or nighttime $T_{\mathrm{mean}}$), BH yields the strongest negative correlation (ρ = −0.33 and −0.27) for the built-up areas. This is primarily caused by the decreased solar altitude in winter accompanied with reduced solar radiation, increased building shadows, and furthermore weakened total surface gain and enhanced convective heat dissipation during the day [10,63,64,65,66,67], which eventually leads to less thermal release at night [4,10]; this effect is reduced in hot summer (ρ = −0.13 and −0.09). This mechanism primarily explains the lower thermal responses of high-rise buildings (LCZs 1, 3.5 and 4) compared to low-rise buildings (LCZs 2.5, 3, and 6) with similar compactness (Figure 6 and Figure 7). Due to the similar impacts during the day and night, the DTR-BH relationship is rather slight (ρ = ~0.00). Note that when non-built-up areas were included in the correlation analysis, ρ became significantly positive in summer (0.35 and 0.27, also reported in [60]) but negligible in winter (0.08 and −0.06), most likely because the overall warming or cooling trend from the vegetated to built-up surfaces in summer or winter dominated the overall relationship.

Among the five indicators, BSF shows the most important heating effect (0.24 ≤ ρ ≤ 0.41), consistent with previous studies [60,68]: with a larger BSF, more heat is trapped in the canyons and stored by building materials, while less heat is lost due to decreased vertical flux change during the day [69], further leading to higher thermal energy released at night [4,10]. Moreover, a larger BSF is often accompanied with a larger population and more anthropogenic heat release. Considering a stronger heating effect during the day and summer than during the night and winter, diurnal and annual variations (DTR and ATR) are enlarged with BSFs (ρ = 0.27 and 0.32). This mechanism supports higher LST responses of compact built-up zones (LCZs 1–3 and 7) than open built-up zones (LCZs 3.5–6 and 9), with similar BH (Figure 6, Figure 7, Figure 8 and Figure 9).

SVF, closely related to BH, BSF, and building layout, can also regulate LSTs. A higher SVF may be associated with lower BH or BSF—the former generates higher LSTs (especially in winter; positive ρ of 0.16–0.19), while the latter forms lower LSTs (especially in summer; negative ρ of 0.29–0.40), as described above. Moreover, different building typologies (e.g., pavilions, terraces, or courts) may have similar SVFs but rather different thermal patterns [27], demonstrating an insignificant LST–SVF relationship [70]. The large low-rise (LCZ 8) and sparsely built (LCZ 9) areas are also examples of insignificance—both showing SVF ≥ 0.7, they presented the highest and lowest LSTs among the built-up zones, respectively (Figure 6; Figure 7). Therefore, the contribution of SVF is mainly attributed to the relative strength of the above influences [66] under changing space and time.

It is surprising that ISF barely correlates with the LST parameters (ρ = ~0.00). This is because the ISF used for correlation was calculated by 100%–BSF–VSF–WSF–SSF (Section 3.1) at a Landsat pixel scale (i.e., 30 m), resulting in a significant surface heterogeneity (or sharp gradient). For example, a block consists of very low ISF over buildings and very high ISF just near the buildings, and yet the LST characteristic does not change dramatically within the same block. Moreover, low ISF can be associated with high BSF, VSF, or others that have contradictory effects on thermal formation. That is one of the reasons that ISF was treated as the lowest rank among the five indicators during the LCZ classification in this study (Section 3.1). It should be clarified that ISF in the LCZ system is different from the impervious surface area (ISA), an indicator describing the ratio of area covered by buildings and impervious surfaces [5,71]. ISA is often derived by spectral unmixing or subpixel classification [72,73], and reported to be strongly and positively correlated with LSTs via trapping, storing, and releasing heat [5,67,74]. A combination of ISF and BSF is close to ISA by definition, therefore greatly increasing the correlation coefficient (ρ = 0.26–0.49).

Under the same mechanisms as describe above, VSF, BH, and SVF have negative or positive influences on the annual HIF or CIF, while BSF has a reversed impact. However, only part of the spatiotemporal LST and UHI variability can be explained by the five indicators of LCZs, which are especially insufficient in winter. As a matter of fact, a variety of additional factors have impacts on LSTs and UHIs, including albedo, landscape pattern, anthropogenic heat release, elevation, urban size, population density, atmospheric condition, and climatology [5]. Some of them are defined in the LCZ system, which mainly drive the general inter-LCZ differences (e.g., Table 4), while some are not, which induce great intra-LCZ differences (e.g., Figure 7) and inter-city and region differences (e.g., Section 5.2). Therefore, a spatiotemporal analysis of driving mechanisms considering various factors (relating to two-dimension and three-dimension, composition and configuration, and regional and local characteristics) needs to be undertaken comprehensively and systematically, which is currently under preparation by Jingling Quan.

### 5.2. Comparisons with Previous Studies

To enhance the inter-site or inter-city comparability for UHI studies and generalize common findings [13], the results for Beijing in this study was evaluated with respect to those of the previous studies conducted for other cities. Those studies were divided into two categories: remotely sensed LST-based and in situ (fixed-site/mobile), AT-based studies (Table 5 [15,16,17,18,22,25,31,33,41]). The study regions include Asian, European, and North American cities with varied climates (e.g., temperate monsoon, arid desert, and oceanic climates) and population ranging from 0.16 to 24 million; the observation period lasted from one day to over a year. Only the results on the ideal days were included in the comparison. The number of standard LCZ types was 7–18 in each LST-based study, while it was 4–14 (six subclasses) in each AT-based study; this study in Beijing had the most types (18, including 15 standard and 3 supplementary classes) because the study area involved complex morphologies due to a long history and rapid urbanization, and the remotely sensed LSTs provided coverage over the entire study area. This contributed to the assessment of the practical thermal behavior of as many LCZs as possible under the same regional conditions. Previous LST-based studies investigated the inter- or intra- LCZ differences, with 2–8 scenes focusing on the instantaneous, seasonal, or day-night patterns, while the AT-based ones were based on 3–78 ideal days (little wind or precipitation, clear skies), focusing on annual and diurnal patterns. This study, using a spatiotemporal fusion model, built LST datasets (hourly frequency on 29 days) that were comparable to the AT datasets from the time scale or period aspect, with a high spatial resolution (100 m), facilitating the examination of inter- and intra-LCZ variability at multiple (i.e., annual, seasonal, monthly, and diurnal) scales. 

Direct comparisons of the specific results between cities and studies are quite challenging because of the differences in city selection, climate, weather condition, instrument, data quality, observation period, spatiotemporal resolution and frequency, and the nature of LCZs [15,22]. Therefore, general trends, rather than values in degree, were analyzed based on the rank order of LCZs in terms of mean values and deviations of temperatures.

First, the built-up zones were typically warmer than the land cover zones—LCZ 10 (heavy industry), LCZ 7 (lightweight low-rise), and LCZ 8 (large low-rise) were mostly recognized as the warmest due to the large anthropogenic heat release and high fractions of impervious and building surfaces and roofs, followed by LCZs 1–3 (compact high-, mid-, or low-rise), while the vegetated types (LCZs A–D) generated lower temperatures due to evapotranspiration and shade, further underlining the role of vegetation in cooling, which was weakened with the defoliation and crop harvesting (consistent between this study and previous studies, Table 5). Regarding LCZ G (water), in comparison to other built-up types, Cai et al. and Yang et al. reported higher ATs and LSTs at night in Shanghai, Hangzhou, and Nanjing (Yangtze River Delta, south of China), explained by the large heat capacity slowing down the nighttime cooling [22,31]. However, this study showed LCZ G to be consistently cooler in Beijing (north of China, much drier than Yangtze River Delta), although its magnitude of difference from other LCZs was reduced at night due to the smaller cooling rate (reaching a consensus on the lowest DTR of LCZ G with previous studies [38,75]). Similarly, Wang et al. recorded LCZ G as the coolest zone during both the day (10:30 am) and night (10:30 pm) in Las Vegas, an arid city in the United States [33], while the LSTs of LCZ G in the Pearl River Delta (south of China) were shown as moderate at night (10:30 pm) [32]. Therefore, the thermal contrast of LCZ G can also be attributed to different geolocations and surroundings, climates (especially relative humidity), and the surface–air interactions within a diurnal cycle, besides the water properties, which require further investigation.

Second, compact built-up zones were basically warmer than open built-up zones because of a larger amount of heat trapped in the canyons and anthropogenic heat continually produced [68]. Among the standard built-up zones, LCZ 4 (open high-rise) was mostly documented to have lower temperatures during the day due to the open canyons, vegetation, shade, and wind pathways that contribute to heat dissipation [22,30,33,35,64,65,76], while LCZ 3.5 (extremely open high-rise), a supplementary class defined in this study, showed even lower LSTs than LCZ 4 in Beijing (Figure 6). A similar trend was also observed between LCZ 4.5 (extremely open mid-rise) and LCZ 5 (open mid-rise), confirming that a lower building density with more pervious surfaces helps ventilation and temperature cooling [16,31]. However, LCZ 2.5 (extremely compact low-rise) showed lower, rather than higher, LSTs than LCZ 3 (compact low-rise) in this study (Figure 6), owing to the effects of building typology. Specifically, LCZ 2.5 mainly has an aligned-terrace typology within the third loop in Beijing [35], which generates lower temperatures than the pavilion, semi-court, and court typologies [27] that are the primary layout patterns in LCZ 3 in Beijing [35]. Regarding the building height, the ranking of LCZs 1–3 or 4–6 was quite variable among cities. Some showed a warming trend with the building height [18,77], whereas some exhibited a gradual temperature increase from high-rise to low-rise zones during the day but a reversed sequence at night because of a larger cooling rate of the lower building height with higher SVFs [33]. Some reported higher LSTs for mid-rise zones than the high- or low-rise zones during the day and night (this study and [25,41]), due to less shade than in high-rise zones and lower SVFs than in low-rise zones, which hinder surface cooling. Moreover, the sequence of the high-, mid-, or low-rise zones may differ seasonally, as shown in this study (Figure 7). Despite these differences, a similar DTR pattern was derived, i.e., lower or higher DTRs corresponded to high- or low-rise buildings (this study and [16]). Further research, such as numerical model simulations, should be conducted to facilitate the understanding of the comprehensive effects of building height and density (commonly combined with materials) on daily and annual temperature cooling and warming patterns, which would support the three-dimensional land planning, while decreasing UHI effects.

Third, intra-LCZ differences were commonly found in different cities at varied intensities. In general, stronger variability emerged during the summer and the day than during the winter and the night (consistent between this study and previous trends [17,18]). Geletič et al. reported that LCZ 8 had the largest spatial variability attributed to varied thermal properties of the large roof materials in Brno and Prague, Czech Republic [25], while this study found LCZ G with the strongest intra-class variability because of the significantly different thermal behavior of lakes and rivers, as well as different water characteristics (e.g., depth, rate of flow) in Beijing. Except the above mentioned trends, no clear pattern was detectable for intra-LCZ variability in this study and the other studies that were based on realistic measurements. This is because the factors and mechanisms are very complex, including not only the limitations of the LCZ scheme in distinguishing the configurations of elements [27], but also the environmental differences of each LCZ unit, such as the relief, traffic, coast, and adjacent land use [16,18,27], and the uncertainties induced by model errors (e.g., LCZ classification and LST fusion and retrieval), missing data, and weather. Note that the impact of thermal anisotropy [53,54] was suppressed to some extent in this study due to the diurnal modeling and multi-temporal average process that integrated varied viewing geometry and solar angles [25]. The intra-LCZ differences lead to varied UHI intensities when selecting different monitoring samples of the same class, which conflicts with the original purpose of the LCZ design; therefore, deeper examination on the multiple aspects of the intra-LCZ variability is under preparation.

Finally, the LST-based studies (including this one) revealed a higher magnitude of differences among LCZs than the AT-based studies [41], owing to the greater heterogeneity of surface temperatures responding to strong heat flux with larger fluctuations over varied thermal properties of the surface [78,79], particularly at a high spatial resolution. This also led to larger LST variability among LCZs during the day than at night (except in winter) (this study and [32,33]), whereas a contrast day-night pattern emerged for the inter-LCZ differences in ATs, i.e., stronger after sunset when different cooling rates are pronounced and weaker during the daytime when advection is pronounced [18,22]. These diurnal variations in the LST-AT relationship further explain why this study, in terms of LSTs, found that the compact built-up zones (LCZs 1–3) had individually larger DTRs than the open built-up zones (LCZs 4–6) at the same height, while Oke and Stewart found the opposite for the ATs [16], considering that the compact zones are often warmer than the open zones during day and night [17,22,25,41]. Additionally, distinct methodology (i.e., remote sensing over Beijing (this study) versus a model simulation over a simplified city [16]) may also contribute to this disagreement. Even though DTRs have been correlated to factors such as the climate, vegetation fraction (negative), impervious surface fraction (positive), soil moisture (negative), canyon aspect ratio (negative), and synoptic condition (e.g., cloud and precipitation, negative) [2,58,79], examination of their combined impacts is still rare. In particular, limited research has documented DTRs of different LCZs (combination of multiple indicators) from the aspect of LSTs; hence, further comparisons are not available in this study. Note that the remotely sensed LST-based studies cannot be compared equally to the in situ AT-based studies because the LSTs and ATs can be greatly different both in space and time [36], attributed to different driving mechanisms and measurement tools. This study does not attempt to replace the AT-based studies but to provide valuable references for understanding the surface temperature distribution and generation, and more ATs from vehicle traverses, automatic weather stations, or volunteered measurements would be greatly helpful. The LST–AT relationship over LCZs is far more complicated than described above, which requires further research but is beyond the scope of the current study.

Overall, this study integrated the advantages of diversity in LCZ, spatial continuity in temperature, and varied time scales characterizing the annual and diurnal temperature dynamics. The results agree with the previous LST-based and AT-based studies in general; however, some differences were detected due to varied climate features, morphologies, methods, data used, and surface-air interactions in individual cities and studies. Therefore, further tests, model development, and local modifications at different spatial and temporal scales are required.

### 5.3. Implications for Public Health

UHIs lead to environmental changes, such as the regional temperature and precipitation, water and air quality, and vegetation growth [5,8,11,80,81,82], which significantly affect public health, such as the thermal comfort, mental health, direct heat-related mortality, and incidence of climate-mediated and triggered diseases [6,83,84,85,86,87,88,89], particularly for the elderly, outdoor workers, and singles and in population-concentrated areas [83]. Urban forms and functions (e.g., compactness and greenness) are key factors driving the spatiotemporal variation of UHIs [4,5,9,10,11] (as demonstrated in this study), and thus most likely pose significant influences on public health [90]. However, this study does not try to quantify the morphology–health or coverage–health, or LST–health or UHI–health relationships, but only attempts to discover the multi-temporal morphology/coverage–LST/UHI relationship to better guide urban planning and management for minimizing the negative thermal impacts of urbanization that include those on public health.

Results indicate that the compact built-up (LCZs 1–3 and 7) and large low-rise (LCZ 8) types generate the strongest UHIs, which should be paid more attention during the urban planning process, while the sparsely built (LCZ 9) area is the most favorable type, showing the weakest UHIs among the built-up types. Green spaces (LCZs A–D) and water bodies (LCZ G) can mitigate the UHI phenomenon but are less effective during the winter and nighttime. It should be clarified that this study is mainly concentrated on LSTs and surface UHIs rather than ATs and atmospheric UHIs that are widely considered in relation to the health issues. However, given the close interactions between LSTs and ATs, these general findings may also be applied to the outdoor thermal comfort (consistent with [91,92]), but with a stronger intra-LCZ variability due to microclimatic impacts [27]. Studies also reveal that high outdoor temperatures increase indoor temperatures in general [93,94,95]. However, the relationship is very complex and affected by factors such as the type of buildings, socioeconomic status, individual behaviors, neighborhoods, and elevations [93]. Therefore, the findings of this study can only provide ambient temperature conditions at the block level for the indoor thermal comfort estimation.

As a matter of fact, satellite-derived LSTs can be adopted to estimate spatial distributions of ATs using physical or statistical models [26,96,97,98,99,100], which can then be used for assessing their impacts to public health quantitatively. However, this study lacks statistical data on disease incidence and mortality rate occurring in the study area (confidential to publics) to connect LSTs or LST-estimated ATs with actual health outcome data in different urban settings, even though it could provide a deeper understanding of the spatiotemporal influence of urban factors on public health at the local scale.

Furthermore, heat risk may be estimated using the LST and UHI data as a hazard layer and the buildings (e.g., CSM or LCZ map) and demographic and socioeconomic data (e.g., age, education, occupation, income, marital status, life style, and cooling devices) as exposure and vulnerability layers [101,102]. Unfortunately, the demographic and socioeconomic data are only partially available at a district level, which hardly supports this study at a local scale. This is why this study mainly focuses on analyzing the climatic prerequisites [89] in different urban settings, which helps indicate areas of higher or lower heat exposure and potentially refer to areas of higher or lower heat risk when the socioeconomic vulnerability is considered average. For example, compact built-up zones (LCZs 1–3) with the highest HIFs (67.7–77.7%, Figure 11) could increase health threats and overall energy demand due to longer duration of exposure to warm and hot temperatures [103]. This is particularly useful for public health preparedness during the extreme heat events [103].

Finally, several limitations exist when using satellite-derived LSTs for health studies. (1) Satellite-derived LSTs are only proxies for heat exposure rather than actual heat exposure at the height (i.e., 1.5 m above the ground) relevant to human health [103]. This differences are less important over a long time frame and at night but more significant over a short term particularly during the daytime [103] (also discussed in Section 5.2). Therefore, the implications of this study for public health should be restricted to the nighttime findings at the annual, seasonal, and monthly scales. (2) The data availability often limits the use of satellite-derived LSTs, considering the trade-off between spatial and temporal resolutions, cloud contamination, and systematic errors [37]. This is particularly problematic in summer [4,37], when heat exposure is high. Although this study constructed hourly LST data by blending multi-source satellite images, it did not recover cloud-contaminated pixels, and thus only scenes on 29 dates were usable throughout a year, excluding the entire month of August (Table 2). (3) Satellite-derived LSTs have been used to estimate daily maximum and minimum ATs [99,100,104] that health-related exposure studies mainly focus on [105]. However, hour-specific ATs are rarely mapped using LSTs at a high spatial resolution. Considering that some health-related indicators (e.g., blood pressure and pulse rate) are variable at a fine time scale (e.g., hourly) [103], the hourly 100-m LST data of this study could be useful for the hourly estimation of ATs (by integrating time-varying coefficients and predictor-related diurnal temperature parameters) [26], and further correlation with such indicators [103].

## 6. Conclusions

Thermal characteristics of different LCZs were investigated in Beijing, China, at multiple time scales (i.e., annual, seasonal, monthly, and diurnal). A total of 18 LCZs (including 15 standard and 3 supplementary classes) were constructed at the block level using a GIS-based method [35]. Hourly LST datasets with a spatial resolution of 100 m were generated on 29 days in 2017–2018 by blending Landsat, MODIS, and FY-2F LST data [37], based on which annual and diurnal cycle parameters of LSTs, as well as HIF and CIFs, were calculated to quantify the inter- and intra-LCZ differences.

The results show comprehensive information on the multi-temporal thermal behavior of different urban settings, which are mainly associated with the urban structure and surface cover properties. The key findings can be summarized as follows: (1) in general, the warmest zones were identified as the compact and mid or low-rise built-up types with little vegetation (LCZs 7, 8, 2, and 3), while the coolest zones were recorded as the water and vegetated zones (LCZs G, A/B, C, and D); (2) LCZs 9, A/B, C, and D showed a general seasonal pattern with smaller ATRs due to leaf abscission, crop harvesting, and irrigation schedule, while the high-rise built-up zones (LCZs 1 and 4) had higher ATRs owing to the seasonal changes in solar radiation through shade effects and convective heat dissipation ability; (3) each LCZ exhibited the largest and smallest DTR in summer and winter, and the DTR was the highest and lowest for the LCZs 2.5, 3, and 8 and A, B, and G, respectively; (4) both the inter- and intra-LCZ differences were stronger during the summer and the day than during the winter and the night; and (5) HIs were the most frequent summertime and daytime events for most built-up zones, while CIs were more frequent in winter daytime and for the high-rise built-up zones (LCZs 1, 3.5, and 4). These findings provide insight into climate-friendly urban planning towards sustainable city development.

Considering the significant but irregular intra-LCZ variability (e.g., Figure 7), partial explanation of driving mechanisms by the five indicators (Section 5.1), and differences in inter-LCZ pattern from previous studies (Section 5.2), further research may explore the physical mechanisms of the LCZ behavior with advanced numerical models concerning the impacts of regional climates and weather conditions, comprehensive effects of building density and height (and materials and layout), intra-LCZ variability, and surface-air temperature interactions. Uncertainties related to the model errors and missing data also remain open to further research.

## Figures and Tables

**Table 1:** Data used for local climate zone (LCZ) mapping.

Data	Observation Time (YYYY/MM/DD)	Spatial Resolution (m)	Layer/Band/Class
City street map (CSM)	2016/03	Vector→15	Block, building, street, water, green space
Landsat-8 OLI ^1^	2016/05/04	15	Red and near infrared bands
Land cover map	2017	30	Forest, shrubland, cropland, grassland, impervious land, bare land, water, snow/ice

^1^ OLI: Operational Land Imager.

**Table 2:** Data used for land surface temperature (LST) fusion.

Date (YYYY/MM/DD)	Landsat-8 TIRS ^1^	MODIS ^2^	FY-2F ^3^ (h)	Date (YYYY/MM/DD)	Landsat-8 TIRS	MODIS	FY-2F (h)
	Observation Time (hh:mm)				Observation Time (hh:mm)		
2017/07/10	10:54	10:48		2018/01/11	-	11:39,12:33,22:00,01:33	24
2017/07/11	-	10:49,13:28,22:54,02:08	24	2018/01/13	-	11:30,12:27,21:54,02:47	24
2017/09/07	-	11:30,12:28,21:54,02:45	24	2018/02/03	10:54	11:10	
2017/09/12	10:54	11:10		2018/02/05	-	11:32,12:30,21:58,02:51	24
2017/09/18	-	11:08,13:39,21:34,02:25	24	2018/02/12	-	11:39,12:33,22:00,01:19	24
2017/09/20	-	10:59,13:30,21:20,02:13	24	2018/02/16	-	11:17,12:16,21:35,02:34	24
2017/09/28	10:54	11:00		2018/03/02	-	11:30,12:27,21:54,02:44	24
2017/09/29	-	10:51,13:28,22:39,02:08	24	2018/03/25	-	11:33,12:30,21:58,02:52	24
2017/10/05	-	10:42,12:50,22:08,01:30	24	2018/04/08	10:54	10:50	
2017/10/24	-	10:45,13:20,22:49,02:00	24	2018/04/11	-	10:36,13:10,22:39,01:57	24
2017/10/30	10:54	11:49,12:40,22:02,01:24	24	2018/04/16	-	11:00,13:30,21:20,02:17	24
2017/11/04	-	10:27,13:00,22:23,01:42	24	2018/04/27	-	10:37,13:10,22:39,01:57	24
2017/11/08	-	11:42,12:33,22:00,01:20	24	2018/05/23	-	11:19,13:41,21:38,02:34	24
2017/11/11	-	10:31,13:03,22:27,01:48	24	2018/05/28	-	11:33,12:30,21:59,02:37	24
2017/11/15	10:54	10:51		2018/05/31	-	10:30,13:00,22:25,01:43	24
2017/12/01	10:54	11:45,12:40,22:02,01:24	24	2018/06/20	-	11:41,12:33,22:01,01:20	24
2017/12/17	10:54	11:48,12:40,22:02,01:24	24	2018/06/27	10:54	11:36,12:43,22:06,01:24	24
2017/12/21	-	11:23,12:23,21:40,02:38	24				
	**Spatial resolution (m)**				**Source**		
	100	1000	5000		USGS ^4^	EOSDIS ^5^	NSMC ^6^

^1^ TIRS: Thermal Infrared Sensor. ^2^ MODIS: Moderate Resolution Imaging Spectroradiometer. ^3^ FY-2F: FengYun-2F. ^4^ USGS: United States Geological Survey, http://earthexplorer.usgs.gov/. ^5^ EOSDIS: Earth Observing System Data and Information System, http://earthdata.nasa.gov. ^6^ NSMC: National Satellite Meteorological Center, http://satellite.nsmc.org.cn.

**Table 3:** Parameters used for thermal analysis of local climate zones (LCZs).

Parameter	Definition and Calculation
Daily/daytime/nighttime $T_{\mathrm{mean}}$	Average of LSTs during a day/08:00–16:00/20:00–04:00
Annual/monthly $T_{\mathrm{mean}}$	Average of daily $T_{\mathrm{mean}}$ in one year/month
Hourly $T_{\mathrm{mean}}$	Average of LSTs at a certain hour in one year
Annual temperature range (ATR)	Difference between the maximum and minimum daily $T_{\mathrm{mean}}$ in one year
Daily $T_{\max}$/$T_{\min}$	Maximum/minimum LST during 24 hours
Daily $t_{\max}$/$t_{\min}$	Time when daily $T_{\max}$/$T_{\min}$ occurs
Diurnal temperature range (DTR)	Difference between daily $T_{\max}$ and $T_{\min}$
Heat/cool island frequency (HIF/CIF)	Rate at which a heat/cool island with a certain intensity occurs during the year/season/daytime/nighttime

**Table 4:** Spearman’s rank correlation coefficients (ρ) between ten thermal parameters and five morphological and coverage indictors ^1^.

Parameter	SVF ^2^	BH ^3^	BSF ^4^	VSF ^5^	ISF ^6^
Annual $T_{\mathrm{mean}}$	−0.12	−0.26 (+0.16)	+0.24	−0.32	+0.03 (+0.32)
Annual temperature range (ATR)	−0.41	+0.10 (+0.31)	+0.32	−0.52	+0.03 (+0.40)
Summer daytime $T_{\mathrm{mean}}$	−0.40	−0.13 (+0.35)	+0.41	−0.49	+0.01 (+0.49)
Winter daytime $T_{\mathrm{mean}}$	+0.19	−0.33 (−0.08)	+0.01	+0.21	+0.00 (+0.03)
Summer nighttime $T_{\mathrm{mean}}$	−0.29	−0.09 (+0.27)	+0.31	−0.35	−0.00 (+0.36)
Winter nighttime $T_{\mathrm{mean}}$	+0.16	−0.27 (−0.06)	+0.01	+0.19	−0.01 (+0.01)
Summer diurnal temperature range (DTR)	−0.29	−0.03 (+0.24)	+0.27	−0.37	+0.02 (+0.36)
Winter DTR	+0.08	+0.00 (−0.07)	−0.06	+0.02	+0.07 (+0.03)
Annual heat island frequency (HIF)	−0.18	−0.25 (+0.20)	+0.27	−0.32	+0.02 (+0.32)
Annual cool island frequency (CIF)	+0.14	+0.24 (−0.16)	−0.22	+0.26	−0.03 (−0.26)

^1^ All values are significant at the 0.01 level. ^2^ SVF (sky view factor) here refers to the ground SV. ^3^ BH represents building height, where values in the brackets were obtained when non-built-up areas were included in the correlation analysis. ^4^ Building surface fraction. ^5^ Vegetation surface fraction. ^6^ ISF represents impervious surface fraction, where values in the brackets were obtained when ISF and BSF were combined in the correlation analysis.

**Table 5:** Comparisons of this study to previous studies. The “H1–H4 and C1–C4” denote the first–fourth hottest and coldest LCZs, respectively. The “x” indicates that the corresponding LCZ type was not included in the study.

Category		Remotely Sensed LST-Based Studies									In situ air Temperature (AT)-Based Studies					
Source/Reference		This Study	[41]	[25]	[31]	[31]	[33]		[33]		[15]	[16]	[17]	[18]	[22]	
Study area	City Country	Beijing China	Szeged Hungary	Prague and Brno Czech Republic	Shanghai China	Hangzhou China	Phoenix USA		Las Vegas USA		Dublin Ireland	Vancouver Canada	Nancy France	Szeged Hungary	Nanjing China	
	Latitude Longitude	40°N 116°E	46°N 20°E	50°N and 49°N 14°E and 16°E	30°N 121°E	29°N 119°E	33°N 112°W		36°N 115°W		54°N 7°E	49°N 123°E	49°N 6°E	46°N 20°E	32°N 119°E	
	Population (million)	22	0.16	1.3 and 0.4	24	9.8	1.5		0.5		1.2	2	0.3	0.16	8.3	
Data	Acquisition method	Landsat, MODIS ^1^ and FY ^2^ fusion	Airborne thermal camera	Landsat and ASTER ^3^	ASTER	ASTER	ASTER		ASTER		Fixed sites and mobile	Mobile	Mobile	Fixed sites	Fixed sites	
	Time period (number of days)	1 year, hourly (29)	Summer night (2)	Spring, summer and autumn day (8 for each city)	Summer and autumn night (2)	Summer and autumn night (2)	Spring day and night (2)		Spring day and summer night (2)		Summer (3)	Spring (4)	Summer (9–17)	1 year (32)	>1 year (78)	
Result	Number of LCZs (sub-/supplementary class)	18 (3)	7 (0)	15 (0)	17 (0)	17 (0)	14 (0)		14 (0)		4 (0)	8 (1)	5 (0)	7 (0)	14 (6)	
	Annual/seasonal pattern	Y	N	Y	N	N	N		N		N	N	N	Y	Y	
	Diurnal pattern	Y (diurnal parameter)	N	N	N	N	Y (day-night)		Y (day-night)		N	Y (DTR ^4^)	Y (day-night)	Y	Y	
	Period of statistics	Annual	Night	Day	Night	Night	Day	Night	Day	Night	Night	Night	Night	Day	Day	Night
	LCZ 1		x	x	H3	H2	x	x	x	x	x	H1	x	x	x	x
	LCZ 2	H3	H1	H3			x	x	x	x	H1	x	H1	H1	H3	H2
	LCZ 3	H4	H3	H2			x	x	x	x	H2	x	x	H1	x	x
	LCZ 4		x			H4		H2		H2	x	H2	x	x	C4	H4
	LCZ 5		H4							H3	x	x	H2	H2	H4	C3
	LCZ 6		C3								H3	C4	H4	H4	x	x
	LCZ 7	H1	x	x					H1		x	x	x	x	x	x
	LCZ 8	H2	H2	H4			H2	H3	H2	H1	x	H3	H3	H3	H1	x
	LCZ 9		C2			C1		C2			x	H4	x	C2		C1
	LCZ 10	x	x	H1	H4	H3		H4		H4	x	x	x	x	H2	H1
	LCZ A	C2	C1	C2	C2	C2	C4	C3	C4		x	C2	x	x	C1	C3
	LCZ B		x	C3			C3		C3	C1	x	C3	x	x		C3
	LCZ C	C3	x	C4	C1	C4	H4		H3	C3	x	x	x	x	x	x
	LCZ D	C4	x		C3		C2	C1	C2	C2	C1	C1	C1	C1	C3	C2
	LCZ E		x		H2		H1	H1			x	x	x	x	x	x
	LCZ F		x		C4	C3	H3		H4	C4	x	x	x	x	x	x
	LCZ G	C1	x	C1	H1	H1	C1	C4	C1		x	x	x	x	C2	H3

^1^ MODIS: Moderate Resolution Imaging Spectroradiometer. ^2^ FY: FengYun. ^3^ ASTER: Advanced Spaceborne Thermal Emission and Reflection Radiometer. ^4^ DTR: diurnal temperature range.

## Appendix A

**Table A1:** Definition and indicators of each local climate zone (LCZ) [13,35]. The indicators used in this study include sky view factor (SVF), building height (BH), building surface fraction (BSF), pervious surface fraction (PSF), and impervious surface fraction (ISF).

LCZ	Indicators	LCZ	Indicators
1 Compact high-rise	SVF: 0.2–0.4 BH(m): ≥25 BSF(%): 40–60 PSF%): <10 ISF(%): 40–60	8 Large low-rise	SVF: ≥0.7 BH(m): 3–10 BSF(%): 30–50 PSF(%): <20 ISF(%): 40–50
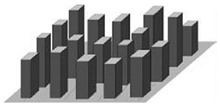
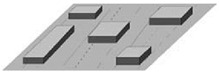
2 Compact mid-rise	SVF: 0.3–0.6 BH(m): 10–25 BSF(%): 40–70 PSF(%): <20 ISF(%): 30–50	9 Sparsely built	SVF: ≥0.8 BH(m): 3–10 BSF(%): 10–20 PSF(%): 60–80 ISF(%): <20
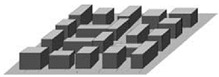
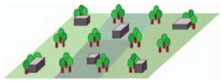
2.5 ^1^ Extremely compact low-rise	BH(m): 4–10 BSF(%): ≥70	10 Heavy industry	SVF: 0.6–0.9 BH(m): 5–15 BSF(%): 20–30 PSF(%): 40–50 ISF(%): 20–40
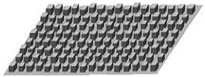
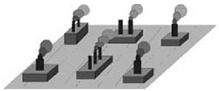
3 Compact low-rise	SVF: 0.2–0.6 BH(m): 3–10 BSF(%): 40–70 PSF(%): <30 ISF(%): 20–50	A Dense trees	SVF: <0.4 BH(m): 3–30 BSF(%): <10 PSF(%): ≥90 ISF(%): <10
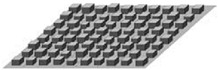
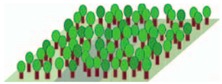
3.5 ^1^ Extremely open high-rise	BH(m): ≥25 BSF(%): 10–20	B Scattered trees	SVF: 0.5–0.8 BH(m): 3–15 BSF(%): <10 PSF(%): ≥90 ISF(%): <10
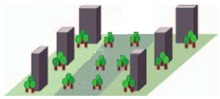
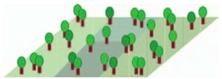
4 Open high-rise	SVF: 0.5–0.7 BH(m): ≥25 BSF(%): 20–40 PSF(%): 30–40 ISF(%): 30–40	C Bush, scrub	SVF: 0.7–0.8 BH(m): <2 BSF(%): <10 PSF(%): ≥90 ISF(%): <10
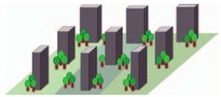
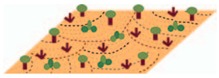
4.5 ^1^ Extremely open mid-rise	BH(m): 10–25 BSF(%): 10–20	D Low plants	SVF: ≥0.9 BH(m): <1 BSF(%): <10 PSF(%): ≥90 ISF(%): <10
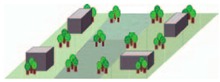
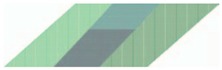
5 Open mid-rise	SVF: 0.5–0.8 BH(m): 10–25 BSF(%): 20–40 PSF(%): 20–40 ISF(%): 30–50	E Bare rock/paved	SVF: ≥0.9 BH(m): <0.25 BSF(%): <10 PSF(%): <10 ISF(%): ≥90
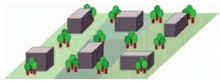
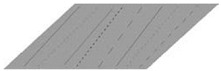
6 Open low-rise	SVF: 0.6–0.9 BH(m): 3–10 BSF(%): 20–40 PSF(%): 30–60 ISF(%): 20–50	F Bare soil or sand	SVF: ≥0.9 BH(m): <0.25 BSF(%): <10 PSF(%): ≥90 ISF(%): <10
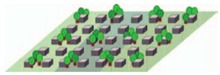
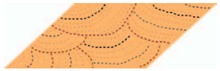
7 Lightweight low-rise	SVF: 0.2–0.5 BH(m): 2–4 BSF(%): 60–90 PSF(%): <30 ISF(%): <20	G Water	SVF: ≥0.9 BH(m): - BSF(%): <10 PSF(%): ≥90 ISF(%): <10
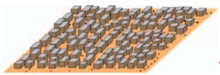
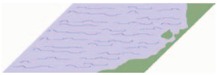

^1^ Supplementary classes defined in this study and classified merely by BH and BSF. LCZ 2.5 (extremely compact low-rise) represents extremely dense mix of low-rise buildings on impervious cover; LCZ 3.5 (extremely open high-rise) represents extremely open arrangement of tall buildings on abundant pervious cover; LCZ 4.5 (extremely open mid-rise) represents extremely open arrangement of mid-rise buildings on abundant pervious cover.

## Appendix B

The single-channel algorithm (used in this study) was developed by Jimenez-Munoz et al. [45,46]:

(A1) $$T=\frac{T_{B}^{2}}{K_{2}L_{\lambda }}\left[\frac{1}{\varepsilon }\left(\psi_{1}L_{\lambda }+\psi_{2}\right)+\psi_{3}\right]+T_{B}^{}-\frac{T_{B}^{2}}{K_{2}}$$

where T is the surface temperature, $T_{B}$ is the at-sensor brightness temperature in Kelvin, $K_{2}$ equals 1321.08 K, $L_{\lambda }$ is the top of the atmosphere (TOA) spectral radiance (unit: W/(m^2^srμm)), equal to $DN_{10}\times 3.3420\times 10^{-4}+0.1-0.29$ ($DN_{10}$ is the digital number of Landsat-8 band 10), ε is the surface emissivity, and $\psi_{1},\psi_{2},\psi_{3}$ are three atmospheric functions.

The brightness temperature $T_{B}$ was calculated according to the calibration parameters provided in the metadata of Landsat images:

(A2) $$T_{B}=\frac{K_{2}}{\ln\left(K_{1}/L_{\lambda }+1\right)}$$

where $K_{1}$ = 774.89 W/(m^2^srμm).

The surface emissivity ε was determined based on the normalized difference vegetation index (NDVI) according to [106]:

(A3) $$\varepsilon =\left\{ \begin{array}{l} 0.9925,\;NDVI\leq 0\\ 0.9230,\;0<NDVI\leq 0.15\\ 1.0094+0.047\ln\left(NDVI\right),\;0.15<NDVI\leq 0.727\\ 0.9860,\;NDVI>0.727 \end{array}\right.$$

where NDVI is derived from the corresponding Landsat-8 OLI data after atmospheric correction (the atmospheric profile parameters were estimated from the NASA Atmospheric Correction Parameter Calculator, https://atmcorr.gsfc.nasa.gov/).

The three atmospheric functions $\psi_{1},\psi_{2},\psi_{3}$ were determined statistically by the water vapor content (obtained from the MODIS water vapor products, http://earthdata.nasa.gov) according to a previous study [45]:

(A4) $$\left[\begin{array}{c} \psi_{1}\\ \psi_{2}\\ \psi_{3} \end{array}\right]=\left[\begin{array}{ccc} 0.04019 & 0.02916 & 1.01523\\ -0.38333 & -1.50294 & 0.20324\\ 0.00918 & 1.36072 & -0.27514 \end{array}\right]\left[\begin{array}{c} \omega^{2}\\ \omega \\ 1 \end{array}\right]$$
